# Site-Specific Lipidomic Signatures of Sea Lettuce (*Ulva* spp., Chlorophyta) Hold the Potential to Trace Their Geographic Origin

**DOI:** 10.3390/biom10030489

**Published:** 2020-03-23

**Authors:** Elisabete da Costa, Fernando Ricardo, Tânia Melo, Renato Mamede, Maria H. Abreu, Pedro Domingues, M. Rosário Domingues, Ricardo Calado

**Affiliations:** 1 Mass Spectrometry Centre, LAQV-REQUIMTE, Department of Chemistry, University of Aveiro, Santiago University Campus, 3810-193 Aveiro, Portugal; elisabetecosta@ua.pt (E.d.C.); taniamelo@ua.pt (T.M.); p.domingues@ua.pt (P.D.); mrd@ua.pt (M.R.D.); 2 ECOMARE, CESAM, Department of Chemistry, University of Aveiro, Santiago University Campus, 3810-193 Aveiro, Portugal; 3 ECOMARE, CESAM, Department of Biology, University of Aveiro, Santiago University Campus, 3810-193 Aveiro, Portugal; fafr@ua.pt (F.R.); renatomamede@ua.pt (R.M.); 4 ALGAplus- Production and Trading of Seaweed and Derived Products Ltd., 3830-196 Ílhavo, Portugal; helena.abreu@algaplus.pt

**Keywords:** Green seaweeds, macroalgae, traceability, lipids, plasticity

## Abstract

The wild harvest and aquaculture of *Ulva* spp. has deserved growing attention in Europe. However, the impact of geographical origin on the biochemical composition of different species and/or strains is yet to be described in detail. Hence, the present study aimed to detect the variability of the lipidome of different species and/or strains of *Ulva* originating from different geographic locations. We hypothesized that lipidomic signatures can be used to trace the geographic origin post-harvesting of these valuable green seaweeds. *Ulva* spp. was sampled from eight distinct ecosystems along the Atlantic Iberian coast and *Ulva rigida* was sourced from an aquaculture farm operating a land-based integrated production site. Results showed significant differences in the lipidomic profile displayed by *Ulva* spp. originating from different locations, namely, due to different levels of polyunsaturated betaine lipids and galactolipids; saturated betaine lipids and sulfolipids; and some phospholipid species. Overall, a set of 25 site-specific molecular lipid species provide a unique lipidomic signature for authentication and geographic origin certification of *Ulva* species. Present findings highlight the potential of lipidome plasticity as a proxy to fight fraudulent practices, but also to ensure quality control and prospect biomass for target bioactive compounds.

## 1. Introduction

*Ulva* spp. are popularly known as “Sea Lettuce” and belong to family Ulvaceae of phylum Chlorophyta. These green seaweeds are known to be opportunistic and highly resilient, occurring in intertidal and subtidal coastal areas, also being able to thrive in freshwater, estuarine environments and even contaminated sites (e.g., sewage sources) [1].

Fresh biomass of *Ulva* spp. consists mainly of water, around 78–80%, and display interesting levels of nutritional elements, such as proteins, lipids, carbohydrates, vitamins and minerals, for human consumption [2,3]. While *Ulva* spp. biomass is often sourced from green tides and used in high volume/low added value markets (e.g., plant and animal care), many of those same species contain commercially valuable components that are now being targeted by high value industries of cosmetics, pharmaceuticals, and miscellaneous chemical applications, along with premium food markets [4,5,6]. For those markets, however, sourcing biomass from aquaculture operations is preferred. The bioprospecting of *Ulva* species has already yielded several new natural products, whose full biotechnological potential is yet to be entirely unraveled [3,7]. The chemical composition of *Ulva* spp. was found to be dependent upon intrinsic (life cycle, phylogeny) and extrinsic factors (e.g., temperature, salinity, nutrients, and, of course, geographical origin) [8,9,10,11]. A viable solution to reduce the impact of biotic/abiotic factors on its composition can be the use of standardized land-based cultivation systems [12,13,14]. These systems also provide the opportunity to produce traceable and replicable quality seaweeds consistently throughout the year, quite important issue particularly when considering *Ulva* spp. as a source of high value-added products with bioactive properties. The ever-growing demand for seafood is promoting the development of aquaculture systems that can meet strict quality standards. The production of *Ulva* species has been found to be higher when grown in tanks rather than at sea, due to the size and morphology of algae itself, along with the lack of competition in batch cultivation, and, when integrated with animal aquaculture, with a non-limiting availability of nutrients [12]. Today, most of the land-based aquaculture operations of *Ulva* spp. is done by clonal propagation of multiple strains, normally collected in the vicinity of farming sites [15]. Species and/or strains selection of *Ulva* spp. can be done for optimizing specific traits, as biomass yield or biochemical composition which can boost its aquaculture profitability. The use of traceability tools for authentication and geographic origin certification of these crops is therefore paramount to differentiate farmed seaweeds, enhance commercial value, as well as expose illegal harvesting and fight fraudulent practices [16]. Food safety alerts together with growing consumer awareness reinforce the need for food traceability. In marine organisms, lipid analysis based on their fatty acids profile has been considered as a reliable tool to trace their geographic origin [17,18]. In seaweeds, lipids are essential structural components of cells and chloroplast membranes, play an important role in several physiological pathways and in the supply of metabolic energy, and have been successfully used as markers to identify contrasting ecological and environmental conditions [19,20,21,22]. Lipidomic signatures are shaped by different extrinsic factors and, as such, can be useful to discriminate species and/or strains originating from distinct harvesting and/or production locations [20]. Lipids from *Ulva* spp. account for 0.3–1.6% of its dry weight (DW) [23] and include mainly galactolipids, sulfolipids, phospholipids, and betaine lipids [24,25,26]. These polar lipids are important to maintain membrane fluidity that adjust to physicochemical conditions of each particular environment [27,28,29] and are the main carrier forms of omega 3 fatty acids and comprise molecules with valuable bioactivities [20,30,31,32,33]. Polar lipids can be used as a tool to address identity and traceability of seaweeds, thereby enhancing their value for food/feed and other industrial applications. Thus, molecular lipid species profiling—of the whole lipidome or per lipid class—may be helpful to trace the geographic origin of wild and/or cultivated *Ulva* species and/or strains and contribute to *Ulva* biomass valorization.

The present work aimed to investigate the potential of lipidomic signatures in wild and farmed *Ulva* spp. along the Atlantic Iberian coast to trace their place of origin, origin effect on lipids quality and on bioactive prospection. The lipidomic signatures of *Ulva* species originating from nine different geographical origins were monitored and a putative set of molecular lipid species was pinpointed holding the greatest potential to reliably trace and monitor the biomass value of *Ulva* species to its harvesting and/or production location.

## 2. Materials and Methods

### 2.1. Reagents

The HPLC-grade dichloromethane, methanol, and acetonitrile were purchased from Fisher Scientific Ltd. (Loughborough, UK). All other reagents were purchased from major commercial sources. Milli-Q water (Synergy, Millipore Corporation, Billerica, MA, USA) was also used in the present work.

Polar lipids internal standards dimyristoyl phosphatidylcholine (dMPC), dimyristoyl phosphatidylethanolamine (dMPE), lysophosphatidylcholine (19:0 LPC), dipalmitoyl phosphatidylinositol (dPPI), dimyristoyl phosphatidylglycerol (dMPG), dimyristoyl phosphatidylserine (dMPS), tetramyristoyl cardiolipin (tMCL), sphingomyelin (17:0 SM (d18:1/17:0)), dimyristoyl phosphatidic acid (dMPA), and N-heptadecanoyl-D-erythro-sphingosine (Cer (d18:1/17:0)) were purchased from Avanti Polar Lipids, Inc. (Alabaster, AL, USA).

### 2.2. Sampling and Storage

**Figure 1:**
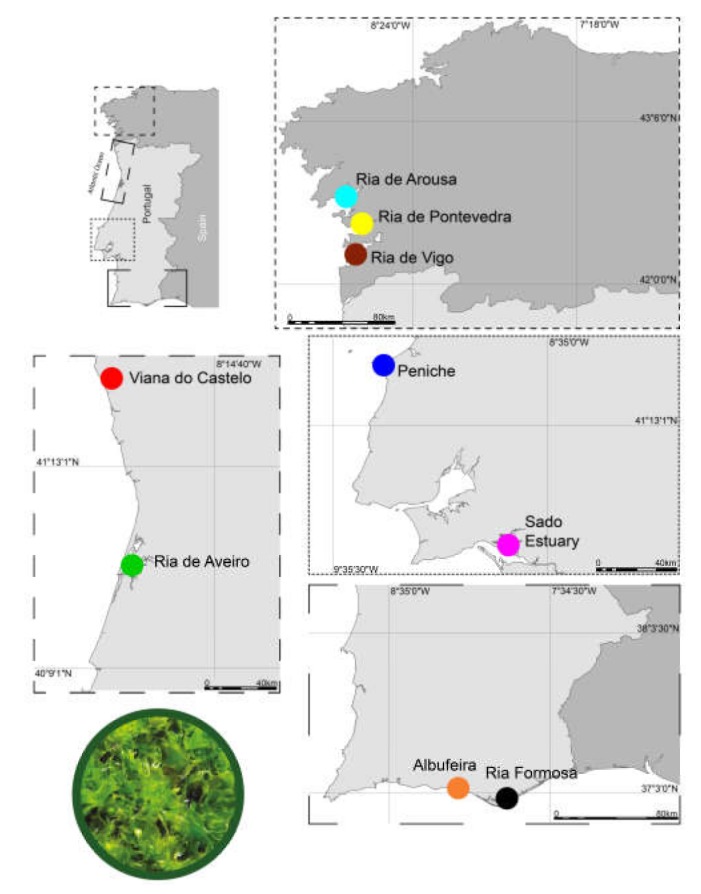
Sampling locations of *Ulva* spp. along the Atlantic western and southwestern Iberian coast: Albufeira (Al), Peniche (Pe), Ria de Arousa (RAr), Ria de Aveiro (RAv), Ria Formosa (RF), Ria de Pontevedra (RP), Ria de Vigo (RV), Sado Estuary (SE), and Viana do Castelo (VC).

Samples of *Ulva* spp. were collected during the summer of 2018 from nine locations along the Atlantic western and south-western Iberian coast (Figure 1): Albufeira (Al: 37°05′21.20′′ N, 8°11′32.70′′ W, water average temperature 19.4 ± 2.1 °C), Peniche (Pe: 39°21′54.50”N, 9°22′20.30”W, water average temperature 18.6 ± 2.2 °C), Ria de Arousa (RAr: 42°30′28.30”N, 8°49′14.10′′ W, water average temperature 17.3 ± 2.1 °C), Ria de Aveiro (RAv: 40°36′46.30′′ N, 8°40′24.70′′ W, ALGAplus, average temperature 22.9 ± 0.8 °C), Ria Formosa (RF: 37°00′23.20′′ N, 7°59′28.40′′ W, water average temperature 21.7 ± 1.3 °C), Ria de Pontevedra (RP: 42°20′25.20”N, 8°45′18.20”W, water average temperature 17.3 ± 2.1 °C), Ria de Vigo (RV: 42°12′00.20′′ N, 8°48′03.20′′ W, water average temperature 17.3 ± 2.1 °C), Sado Estuary (SE: 38°28′01.90′′ N, 8°59′25.10′′ W, water average temperature 19.2 ± 2.1 °C) and Viana do Castelo (VC: 41°41′45.70′′ N, 8°51′08.90′′ W, water average temperature 17.3 ± 2.3 °C). Five specimens were hand-collected from the wild on each sampled location, except those from Ria de Aveiro which were supplied by a commercial farm (ALGAPlus Lda.) producing *Ulva rigida* using an open land-based integrated multi-trophic aquaculture (IMTA) system (9 locations × 5 replicates = 45 samples). The IMTA system operates in the semi-intensive way, water flows in one direction only through a Ria de Aveiro channel surrounding the farm, enters the production system at each high tide. Water passes through fishponds and flows back to the lagoon at low tides. *Ulva rigida* was cultivated in tanks at average water temperature (°C) and salinity (PSU) 22.9 ± 0.8 °C and 36.4 ± 0.3 in the summer over 10 days, collected, cleaned to remove epiphytes, and oven dried at 25 °C (moisture below 12%, *w*/*w*). All algal samples from the wild were immediately stored in aseptic bags and transported to the laboratory. After sampling, *Ulva* spp. were washed repeatedly under running tap water and then with distilled water to remove epiphytes and impurities. After washing, all samples were freeze dried and maintained at −80 °C until further analysis.

The identification of species within genus *Ulva* has long been recognized as an extremely challenging task, with these green seaweeds displaying a remarkable level of morphological plasticity (e.g., multiple strains occurring within the same species) and cryptic speciation being commonly recorded [34]. These features commonly impair an accurate taxonomic classification of sampled material and cause undesirable taxonomic confusion. These issues of taxonomic inaccuracy are particularly relevant when targeting species that may yield valuable natural products [34]. In this way, samples collected in the present study were termed as *Ulva* species. Fragments were preserved for future species identification once the taxonomic issues of these genus are clarified and robust DNA barcoding protocols are made available for family Ulvaceae. Moreover, the present study was framed within an industry-driven perspective, with European algal farmers and harvesters acknowledging that it will be virtually impossible to safeguard that any given batch of farmed or harvested sea lettuce will be solely composed by a single species (or in other words a “pure” batch of *Ulva* A or *Ulva* B). Currently, European algal farmers and harvesters claim that traded batches of sea lettuce should be simply labelled as *Ulva* spp. or only as sea lettuce (with no scientific name assigned to account for possible taxonomic revisions that may exclude commercially relevant species from genus *Ulva*). As such, already forecasting this scenario, the authors decided to analyze the samples collected as a whole, considering them as representative of the lipid diversity hosted by the batch of seaweeds being cultured or growing naturally in different locations, regardless of featuring solely one species or a mix of sea lettuce species. This rationale is therefore aligned with the needs from the European algal farming and harvesting industry that already revealed that genotyping each batch of *Ulva* being traded will simply take away the profitably of commercially exploring these seaweeds.

### 2.3. Biochemical Composition

The moisture content of freeze-dried samples was determined on a dry basis (%DW) by the weight loss of 250 mg in an oven at 105 °C for 15 h (five replicates). The difference between the initial weight and the final weight, which is equivalent to the moisture vaporized, was used to calculate the percentage of moisture drying. The ash content was obtained after submitting the dried samples to ashing in a muffle furnace at 575 °C for 6 h (%DW). The protein fraction was calculated from elemental N (Leco Truspec-Micro CHNS 630-200-200 elemental analyzer) using the nitrogen–protein conversion factor of 6.25. Lipid content was determined according with the methodology described in the following section. Total carbohydrate and other constituents were determined by difference.

### 2.4. Lipid Extraction

Total lipid extraction was performed by adding 3.75 mL of methanol/dichloromethane (2:1, per volume) to samples of 250 mg of freeze-dried seaweed (five replicates). The mixture was homogenized and incubated on ice on a rocking platform shaker (Stuart Scientific STR6, Bibby, UK) for 2 h and 30 min. The mixture was centrifuged at 392 rcf for 10 min (Pro-Analytical series, UK). The organic phase was collected, and 1.25 mL of dichloromethane and 2.25 mL of water were added for phase separation. The biomass residue was re-extracted three times with 1 mL of MeOH and 0.5 mL of CHCl_3_. Water was added to the collected organic phases, followed by centrifugation for 10 min, and the organic (lower) phase was recovered. Solvents were dried under a stream of nitrogen gas. The total lipid extract content was estimated by gravimetry (transference of extracts to dried and weighed amber vials). Lipid extracts were stored at −20 °C before analysis by LC–MS.

### 2.5. Ultra High-Performance Liquid Chromatography-Mass Spectrometry

Ultimate 3000 Dionex ultra high-performance liquid chromatography (UHPLC) system (Thermo Fisher Scientific, Bremen, Germany) with an autosampler coupled online to the Q-Exactive mass spectrometer with Orbitrap technology (Thermo Fisher Scientific, Bremen, Germany). The solvent system consisted of two mobile phases as follows: mobile phase A (acetonitrile:methanol:water 50:25:25 (per volume) with 2.5 mM ammonium acetate) and mobile phase B (acetonitrile:methanol 60:40 (per volume) with 2.5 mM ammonium acetate). Initially, 10% of mobile phase A was held isocratically for 2 min, followed by a linear increase to 90% of A within 13 min and a maintenance period of 2 min, returning to the initial conditions in 8 min and held for more 10 min. A volume of 5 µL of each sample containing an amount equivalent to 5 µg of lipid extract, a volume of 4 μL of phospholipid standards mix (dMPC—0.02 μg, dMPE—0.02 μg, SM (d18:1/17:0)—0.02 μg, 19 Lyso PC—0.02 μg, dPPI—0.08 μg, CL(14:0)_4_—0.08 µg, dMPG—0.012 μg, dMPA—0.08 μg, Cer (d18:1/17:0)—0.04 μg, dMPS—0.04 μg) and 91 µL of eluent B was introduced into a microbore Ascentis^®^ Si column (10 cm × 1 mm, 3 µm, Sigma–Aldrich) with a flow rate of 50 µL min^–1^ and at 35 °C. The mass spectrometer with Orbitrap^®^ technology was operated simultaneously in positive (electrospray voltage 3.0 kV) and negative (electrospray voltage –2.7 kV) modes with a resolution of 70,000 and automatic gain control (AGC) target of 2 × 10^6^, the capillary temperature was 250 °C and the sheath gas flow was 15 U. In MS/MS experiments, a resolution of 17,500 and AGC target of 1 × 10^5^ were used. Cycles consisted of one full-scan mass spectrum and ten data-dependent MS/MS scans were repeated continuously throughout the experiments with the dynamic exclusion of 60 s and intensity threshold of 1 × 10^4^. Normalized collision energy™ (CE) ranged between 20, 25, and 30 eV. Data acquisition was carried out using the Xcalibur data system (V3.3, Thermo Fisher Scientific, USA). The identification of lipid species was performed using LC-MS typical retention time. Accurate mass measurements (≤ 5 ppm) were employed to confirm the elemental composition and analysis of the MS/MS spectra to confirm molecular composition of lipid species data. MS data was acquired in positive ion mode for monogalactosyl diacylglycerol (MGDG), monogalactosyl monoacylglycerol (MGMG), digalactosyl diacylglycerol (DGDG), and digalactosyl monoacylglycerol (DGMG) classes, identified as [M + NH_4_]^+^ ions, for diacylglyceryl 3-O-4′-(N,N,N-trimethyl) homoserine (DGTS), monoacylglyceryl 3-O-4′-(N,N,N-trimethyl) homoserine (MGTS), phosphatidylcholine (PC), lyso-phosphatidylcholine (LPC), phosphatidylethanolamine (PE), and lyso-phosphatidylethanolamine (LPE) classes, identified as [M + H]^+^ ions. The MS was acquired in negative ion mode for sulfoquinovosyl diacylglycerol (SQDG), sulfoquinovosyl monoacylglycerol (SQMG), phosphatidylglycerol (PG), lyso-phosphatidylglycerol (LPG), phosphatidylinositol (PI), PE, and LPE classes, identified as [M − H]^−^ ions, while PC and LPC were seen in negative mode, identified as [M + CH_3_COO]^−^ ions. The MS/MS of species-specific ions were analyzed and lipid classes were characterized according to fragmentation patterns, as previously described [24,35].

### 2.6. Data Analysis

The MS raw data were pre-processed by filtering and smoothing, peak detection, peak processing, and assignment against an in-house database by using the software package MZmine 2.39 with a mass tolerance of 5 ppm. Data integration was expressed as normalized data against lipid internal standards of each lipid class. Data were processed by estimation of missing values by replacing it by half of the minimum positive values in original data; data were filtered using median intensity value, glog transformed and autoscaled using the R package Metaboanalyst 4.0 [36,37]. Relative quantitation was performed by exporting integrated peak areas values into a computer spreadsheet (Excel, Microsoft, Redmond, WA). Bar graphs were created using the software GraphPad Prism 5 (Supplementary Materials Figures S1–S5). Resampling simulations were carried out independently by bootstrap, based on 30 theoretical samples of the original set that were used for each simulation. Principal component analysis (PCA) was performed using the R built-in function and ellipses were drawn using the R package, assuming a multivariate normal distribution and a level of 0.95 (Metaboanalyst 4.0). One-way ANOVA (analysis of variance) followed by post-hoc Tukey’s honestly significant difference test (Tukey’s HSD) were performed with R built-in function. *p*-values were corrected for multiple testing using Benjamini–Hochberg false discovery rate (FDR, q-values). Heatmaps were created using the R package using “Euclidean” as clustering distance, and “ward.D” as the clustering method (Metaboanalyst 4.0). The top 25 lipid species was ranked using the lowest q-values along false discovery rate Benjamini–Hochberg procedure and were used to run a new PCA for a complimentary exploratory data analysis. The existence of significant differences (*p*-values < 0.05) between ecosystems was tested for each biochemical parameter and for molecular species through generalized linear models (GLM) using a gamma distribution. Graphics and boxplots were generated using R package ggplot2 [38].

## 3. Results

### 3.1. Biochemical Composition of Ulva spp

Biochemical composition expressed as total lipids, protein, minerals as ash, and carbohydrate and other compounds varied extensively between *Ulva* spp. from different geographical origins (Tables S1 and S2). Total lipids content (%DW) varied significantly (*p* < 0.0001) and ranged from 0.34 ± 0.06% DW (in RF) to 1.77 ± 0.07% DW (in RP) (a 5.2-fold variation in lipid content). No significant differences were found between total lipid content from RF, RV, SE, and RAv specimens, which displayed the lowest lipid content; lipid content in *Ulva* spp. collected in Pe and RP displayed the highest lipid yields (> 1% DW) and did not differ significantly between these two locations. There were also significant differences in protein and ash contents displayed by *Ulva* spp. originating from different locations (Table S2). Protein content ranged from 4.70 ± 0.31% DW (in RV) up to 18.13 ± 1.26% DW (in Pe), while ash ranged between 13.48 ± 0.80%DW (in SE) up to 27.79 ± 2.51% DW (in RAv). Total carbohydrate and other constituents also displayed significant differences across different locations and ranged from 53.36 ± 1.30% DW (in Pe) up to 78.67 ± 0.31% DW (in RF) (Table S1).

### 3.2. Lipidome Plasticity of Ulva spp. from Different Geographic Locations

The lipidomic profile of *Ulva* spp. investigated by hydrophilic interaction liquid chromatography (HILIC)–LC–MS analysis yielded 201 lipid species distributed over galactolipids monogalactosyl diacylglycerol (MGDG), monogalactosyl monoacylglycerol (MGMG), digalactosyl diacylglycerol (DGDG), digalactosyl monoacylglycerol (DGMG); sulfolipids sulfoquinovosyl diacylglycerol (SQDG), sulfoquinovosyl monoacylglycerol (SQMG); phospholipids phosphatidylcholine (PC), phosphatidylethanolamine (PE), phosphatidylglycerol (PG), their lyso lipids, and phosphatidylinositol (PI); and betaine lipids diacylglyceryl 3-O-4′-(N,N,N-trimethyl) homoserine (DGTS) and monoacylglyceryl 3-O-4′-(N,N,N-trimethyl) homoserine (MGTS).

The comparison of the lipid profile of *Ulva* spp. from the nine geographic origins revealed that 23 lipid species were present in specific locations, as summarized in Table 1. The group included four DGMG, three DGDG, two SQDG, three DGTS, one MGTS, five PC, one LPE, three PE, and one LPG species. Among these lipid species, DGMG (16:2) and DGMG (16:3) were exclusively identified in *Ulva* spp. from RV, while DGMG (16:4) was only identified in *Ulva* spp. from RAv.

The relative abundance of lipid species from each class was then compared (bar graphs in Figures S1–S5) and differences were recorded in some classes of specimens originating from different locations. In the class of MGDG, the most abundant lipid species in specimens collected from Pe, RAr, RP, RV, VC was MGDG (34:7); the lipid species MGDG (34:8) was the most abundant in specimens from Al, RF, and SE; and MGDG (36:6) was the most abundant in specimens produced in RAv. In the case of lyso galactolipids, MGMG 16:0 was the most abundant in *Ulva* spp. from Al, RAr, RF and SE while MGMG (16:4) was the most abundant in *Ulva* spp. collected from RAv, Pe, RV, VC, and RP. A more consistent pattern independent of geographic origin was detected for the remaining glycolipids from classes DGDG, DGMG, and SQDG with DGDG 34:2 and DGDG 34:3; DGMG 16:0; and SQDG (34:1) being the most abundant species, respectively. In the classes of phospholipids, PC (30:3) and PI (38:8) were much more abundant in *Ulva* spp. collected from RP while, in the remaining locations, PC (36:2) and PI (34:2) were generally the most abundant species. PG class profile presented a somewhat variable profile across different origins inferred by the abundant PG (34:2) in AL, RAv, RF, and RV; PG (34:4) in PE and VC; and PG (40:6) in RP and RAr. LPC (16:0) and PE (34:2) were reliably the abundant species in the corresponding classes. In betaine lipids, DGTS (34:4) and MGTS (18:4) were generally the most abundant species. Exceptions were registered for DGTS (34:3), the most abundant species in RP; DGTS (32:1), predominant in the cases of RAv and SE; MGTS (16:0), abundant in the lipidomes from *Ulva* spp. collected in Pe, RAv, and SE; and MGTS (18:3), more abundant in the RP.

**Figure 2:**
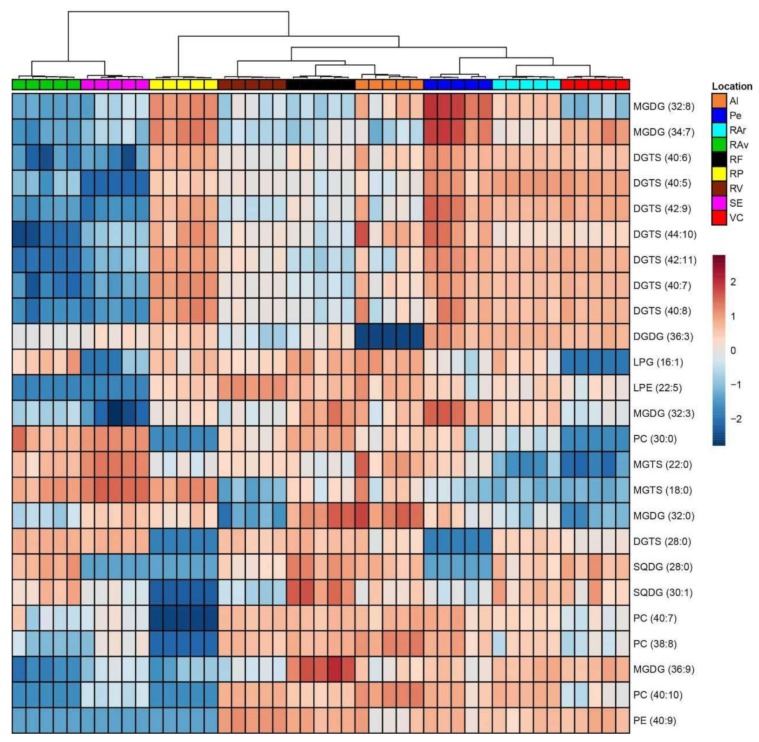
Hierarchical clustering heatmap of lipid species data. Levels of normalized peak area are shown on the color scale, with numbers indicating the fold difference from the mean. The clustering of the sample groups is represented by the dendrogram of the top 25 lipid species displaying the lowest Tukey’s HSD test q-values. Labels of the sites are according to the notation: Albufeira (Al), Peniche (Pe), Ria Arousa (RAr), Ria de Aveiro (RAv), Ria Formosa (RF), Ria de Pontevedra (RP), Ria de Vigo (RV), Sado Estuary (SE), and Viana do Castelo (VC). Albufeira (Al), Peniche (Pe), Ria Arousa (RAr), Ria de Aveiro (RAv), Ria Formosa (RF), Ria de Pontevedra (RP), Ria de Vigo (RV), Sado Estuary (SE), and Viana do Castelo (VC). Labels of lipid species are according to the notation: AAAA (C:N), (AAAA, lipid class; C, total carbon atoms; N, total double bonds of fatty acid substituents).

Data sets of the 201 lipid species were further surveyed for exploratory purposes using PCA. The visualization of samples grouped with location was obtained, but there was not a clear separation between groups (Supplementary Figure S6). The eigenvalues of the two first principal components represented 54.1% (PC1, 31.9%; PC2, 22.3%). Hence, dataset was sorted using the lowest q-values along false discovery rate (one-way ANOVA, post-hoc Tukey’s HSD test and FDR Benjamini and Hochberg procedure) and the top 25 lipid species displaying the lowest q-values were ranked and used to create the heatmap (Figure 2). The top 25 list included five MGDG, one DGDG, two SQDG, four PC, one LPG, one LPE, one PE, eight DGTS, and two MGTS species (Supplementary Information, Table S4). The dendogram showed the separation of the nine locations (Figure 2). The first level of separation was evidenced between specimens from RAv and SE and the remaining origins. The second level of separation distinguished *Ulva* species from RAv, SE, and RP from the six remaining groups. Remaining groups were differentiated in six clusters in the third and fourth levels of separation.

**Figure 3:**
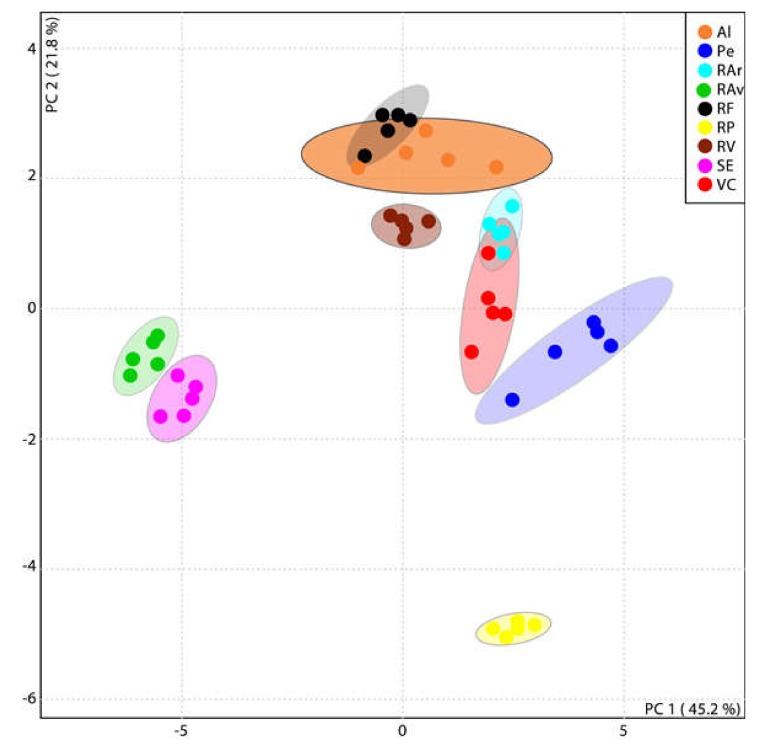
Principal component analysis (PCA) score plot of the top 25 lipid species displaying the lowest Tukey’s HSD test q-values. Albufeira (Al), Peniche (Pe), Ria Arousa (RAr), Ria de Aveiro (RAv), Ria Formosa (RF), Ria de Pontevedra (RP), Ria de Vigo (RV), Sado Estuary (SE), and Viana do Castelo (VC).

A new PCA considering these top 25 lipid species was performed and a new grouping was plotted (Figure 3). The eigenvalues of the PC1 plus PC2 increased, explaining now 67% of total variance of the observations. PCA analysis of the top 25 lipid species LC–MS data set showed seven groups representing each individual location and two overlapped groups: Al and RF (accounting for the southern coast region) and VC and RAr (accounting for the northern coast region). Along axis I (PC1), RP, Pe and RAr-VC groups were positioned closer together and opposite to RAv and SE locations, while along axis II (PC2) RP, Av, SE and Pe were discriminated laying opposite to RV and the RAr-VC and Al-RF groups.

**Figure 4:**
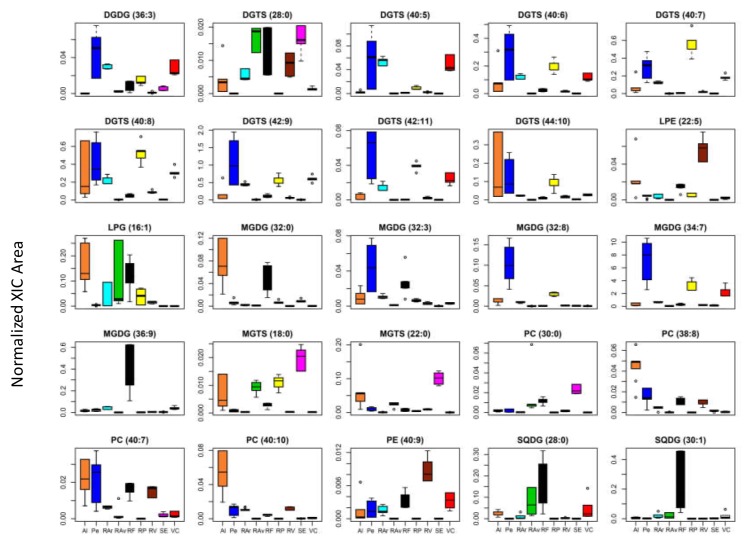
Boxplots of the top 25 lipid species sorted by Tukey’s HSD test displaying the lowest q-values. Normalized areas were obtained from extracted-ion chromatogram (XIC). Albufeira (Al), Peniche (Pe), Ria Arousa (RAr), Ria de Aveiro (RAv), Ria Formosa (RF), Ria de Pontevedra (RP), Ria de Vigo (RV), Sado Estuary (SE), and Viana do Castelo (VC). Labels of lipid species are according to the notation: AAAA (C:N), (AAAA, lipid class; C, total carbon atoms; N, total double bonds of fatty acid substituents).

The normalized areas of the top 25 lipid species are represented in the boxplots of Figure 4 and their contribution for site-specific discrimination of geographic origin was evaluated using univariate pairwise comparison (Supplementary Information, Table S5). Significant levels of variability were obtained, which allowed the identification of specific metabolites as putative candidates for origin discrimination. The molecular composition of these lipid species, assigned by Tandem mass spectrometry (MS/MS) data analysis, is summarized in Table 2. This set of metabolites can be useful to discriminate *Ulva* spp. originating from different geographic locations. Specimens from RV were specifically assigned to that location through higher abundance of phosphatidylethanolamine species LPE (22:5) and PE (40:9) (*p* < 0.0001). *Ulva* spp. originating form Pe were differentiated by higher abundance of MGDG (32:3), MGDG (32:8), MGDG (34:7), DGDG (36:3), DGTS (42:9), and DGTS (42:11) (*p* < 0.0001). Concerning biomass from SE, this was assigned to its specific geographic origin by higher abundance of MGTS (22:0), MGTS (18:0), and PC (30:0) (*p* < 0.0001), while biomass from RAv was assigned through higher abundance of SQDG (28:0) and DGTS (28:0). Specimens originating from RF were particularly differentiated through higher abundance of MGDG (36:9) and SQDG (30:1), while those form Al were specifically assigned by phosphatidylcholine species PC (38:8) and PC (40:10). It is noteworthy to highlight the differentiation between northern group samples, such as RAr, RP, and VC, from other geographic origins, mainly due to the contribution of betaine lipids species containing polyunsaturated fatty acids (16:4, 22:5, 18:2, 18:3, 18:4; *p* < 0.0001). *Ulva* spp. originating from RP could be discriminated through higher abundance of DGTS (40:7) and DGTS (40:8), while phospholipids LPG (16:1), PC (40:7) and betaine lipids DGTS (28:0) allow discrimination among RAr and VC origin of seaweeds.

At molecular level, lipid profiling of lipid class species together with statistical and cluster analysis proved to be effective in the discrimination of *Ulva* specimens per geographical origin.

## 4. Discussion

Lipids are essential molecules that maintain the integrity of the cells and can act as signaling compounds to control vital biological processes and protect organisms from harsh environments [21,39]. Owing to seaweeds’ habitat diversity, lipid composition is influenced by environmental factors, natural and anthropogenic based, that shape algal nutritional composition [28,40]. Thus, lipid biomolecules emerge as important biomarkers of environmental conditions, encouraging their use to trace the geographic origin of wild and/or farmed *Ulva* species. In this scope, the present study demonstrated how lipidomics, framed by a suitable statistical data analysis to cope with lipid variation [41,42], can be a robust tool to trace site-specific lipid signatures from *Ulva* spp. collected in marine and estuarine ecosystems, as well as farmed under controlled conditions in land-based systems. According to nutritional and value-added of these lipid species, *Ulva* specimens can be selected for farming purpose and further utilization for food, feed, nutraceutical or pharma applications.

The first screening performed on *Ulva* spp. from different geographic origins to determine its biochemical composition revealed that the proportion of total lipids (TL) was higher in specimens from northern and center Atlantic coast (RP and Pe, respectively). These locations are both characterized by colder water (average water temperature 17.3 °C and 18.6 °C, respectively, Source: www.seatemperature.org/europe), even during the summer, being Pe more exposed to oceanic winds. In line with this result, the minimum TL content was recorded in specimens harvested in RF, the southernmost location sampled, which occur in warmer waters during the summer (average 21.7 °C, Source: www.seatemperature.org/europe). The highest TL content did not surpass 2% of the whole dried seaweed biomass, agreeing with previous studies performed on *Ulva lobata*, *Ulva* spp., and *Ulva pertusa* during summer [19,26,43,44]. It is generally accepted that temperature strongly affects TL in seaweeds [45,46], although the effect of shifting salinity, pH, light exposure, nutrients availability, among other factors, should not be neglected [39,47]. These findings encouraged the in depth-study of lipid profiles, at a molecular level, to better understand adaptive lipid profiling and biomarkers discovery of *Ulva* spp. between different geographic origins.

The lipidome of all algal specimens surveyed yielded the identification of glycolipids (MGDG, DGDG, SQDG, and their lyso forms MGMG, DGMG, SQMG); phospholipids (PG, PC, PE, PI, and the lyso-forms LPG, LPC, and LPE); and betaine lipids (and lyso-form MGTS). These classes have been already identified in the lipidome of *Ulva rigida* produced on land integrated multi-trophic aquaculture system and *Ulva lactuca* harvested from the Adriatic Sea and the Sea of Japan [24,48,49].

From the 201 lipid species identified in the present study, 178 were common to all specimens from the nine geographic locations surveyed. Nonetheless, it is noteworthy to highlight that some species such as lyso-DGDG (16:x type) were only present on a few specific locations (Table 1), namely DGMG 16:2 and DGMG 16:3 (in specimens from RV) and DGMG 16:4 (in specimens from RAv). To date, not much is known about the role that lyso-galactolipids may play on the ability of seaweeds to cope with environment stress, although lyso-compounds were found to be important in galactolipid turnover and fatty acid re-modelling on salt tolerant plants coping with desiccation [50], as well as in the response of *Arabidopsis* to wounding [50,51]. Specimens originating from RP were distinguished by the lake of some lyso-lipids and phospholipids described in Table 1. The effect of different nutrients in *Ulva* spp. collected from RP has been reported [52] and, in the summer, nutritional limitation such as minimum levels of phosphorus was recorded. It is well described that, in green algae, the regulation of betaine lipids and phospholipids in cell membranes is important [53,54]. As a general rule, betaine lipids are present in the polar lipids of green seaweed and there is an assumption of mutual substitutability of DGTS and PC in membrane lipids [49,55,56].

The clustering of all lipid species data after multivariate analysis of the LC–MS data showed a non-clear distinction between the different geographical origins. However, when solely considering the top 25 most contrasting lipid molecular species (Table 2) a clear distinction of *Ulva* specimens per geographical origin could be achieved (Figure 2). In spite of their short geographic distance *Ulva* originating from RP was clearly separated from specimens collected at RAr and RV (Figure 2). The waste water of industrial and urban origins and high nitrogen inputs have been related to the presence of green patches of Ulvaceae in RP [57]. These anthropogenic factors affecting coastal environments can shape the growth and foster specific biochemical features in the composition of *Ulva* [58], hence supporting the differentiation of specimens originating from RP. The structural details of these metabolites set obtained to discriminate *Ulva* spp. were characterized at molecular level (by LC–MS/MS). Generally, fatty acyl composition of MGDG (containing polyunsaturated C16, C18 and C20 acyl chains); SQDG (containing saturated and unsaturated C16 acyl chains); and PLs and DGTS (containing polyunsaturated C18 and C20) classes is in accordance with expected fatty acid composition of *Ulva* [39,48,59]. However, the differences achieved at lipid species level suggest the important role of the polar lipids MGDG, DGTS and PLs in the adaptive changes of cell membranes to environmental factors characteristic of each specific geographic origin. In respect to MGDG, it is considered to be paramount for structure flexibility of thylakoids membranes when coping with temperature oscillations, specially through the adjustment of the unsaturation level of fatty acids to facilitate membrane deformation (low temperature, increased unsaturation) [48]. The unsaturation/saturation adjustments of DGTS and PC has been well reported and related with adaptative changes of *Ulva* spp. to maintain extraplastidial cell membranes homeostasis [48,49]. This compensatory mechanism relates to the homeoviscous adaptation of cell membranes lipids to maintain the viscosity and the integrity of organelles at different environmental temperatures. Moreover, greater variability between replicates was observed in some of populations, e.g., Al, Pe, and VC. These are marine coastal ecosystems, which are likely more exposed to pronounced shifts in physico-chemical environmental drivers that shape the biochemical profile of seaweeds, namely of those colonizing intertidal regions [60]. Furthermore, samples were collected only in the summer period, a season that has been associated with harsh environmental conditions, such as higher PAR levels and salinity, a set of abiotic factors that strongly influences biosynthesis of lipid species in seaweeds [11,42,60]. Future studies should perform a follow-up analysis of the present data being reported by shedding light on how seasonal shifts experienced by seaweeds can be an advantage or a caveat to use lipid species fingerprints to reliably allocate the geographic origin of *Ulva*. Concerning molecular species-specific relevance for each particular origin differentiation, *Ulva* spp. originating from Pe was differentiated by high unsaturated chloroplastidial MGDG (32:3), MGDG (32:8), MGDG (34:7), and DGDG (36:3), and extraplastidial DGTS (42:9) and DGTS (42:11). Recently, the evaluation of the behavior of membrane lipids from *Ulva lactuca* inhabiting different climatic zones showed that polyunsaturated molecules such as MGDG (34:7), also found in the present study, displayed a sharp increase in colder environments [48]. Moreover, it is noteworthy to highlight the contribution of polyunsaturated betaine lipid species containing 16:4, 22:5, 18:2, 18:3, and 18:4 fatty acids for separate samples originating from northern locations such as RAr, RP, and VC, and remaining geographic origins. The polar head group of DGTS, which is large enough to help maintaining membrane-bilayer homeostasis even at low temperature, was pinpointed as an important feature when adapting to temperature shifts [54]. *Ulva* spp. originating from RP was discriminated by higher contents of DGTS (40:7) and DGTS (40:8), when compared with other locations, while phospholipids LPG (16:1), PC (40:7) and betaine lipids DGTS (28:0) contributed for differentiating RAr seaweeds from those of VC origin. Moreover, LPG 16:1, that differentiated RAr origin, has been claimed to be involved in the repair of thylakoid proteins that have been photochemically damaged [40]. *Ulva* spp. originating from RV was featured by PE species such as LPE (22:5) and PE (40:9). PE changing trends and functions with environmental stressors is still unclear for seaweeds. However, long-chain unsaturated PE species increased in salt tolerant plants to cope with higher salinities while LPE, formed by hydrolysis of PE parent, is considered important in mediating protective mechanisms, such as systemic response to wounding [61].

Concerning biomass from SE, extraplastidial PC (30:0) and lyso betaine lipids ((MGTS (22:0) and MGTS (18:0)) were important to discriminate this geographic origin, while that from RAv was assigned through chloroplastidial SQDG (28:0) and extraplastidial DGTS (28:0). Algae originating from both ecosystems featured saturated lipid species. The saturation level of membrane lipids was considered an important response of seaweeds to cope with warm temperatures [20,48]. Average temperature recorded on both ecosystems was warmer when compared to northern ecosystems (Source: www.seatemperature.org/europe), suggesting that the presence of saturated lipid species may result from combined effects of temperature and other environmental conditions. The salinity level variability of both the Sado Estuary [62] and Ria de Aveiro [63,64] has been studied, both reflecting the influence of existing saltpans. The role of membrane lipids as signaling molecules in response to salinity can herein play a role [19,65]. Salinity-induce shifts in membrane lipids that include changes on the content of lipids, fatty acid unsaturation level, and fatty acid chain length to regulate and reduce the fluidity and permeability of the cell membranes [19,66,67]. Studies on total fatty acids composition exposed to different levels of salinity of *Ulva pertusa* unveiled an increase of the quantitative saturated fatty acids content both under hypo- and hypersalinity conditions [19].

Southern origin specimens, such as those from RF, were particularly differentiated through MGDG (36:9) and SQDG (30:1), while those form Al were specifically assigned by phosphatidylcholine species PC (38:8) and PC (40:10). The main differences between both locations were related to PC lipid species. Both ecosystems are exposed to higher temperatures than other study-sites. Previously, lower nutrient content has been reported in *Ulva* specimens inhabiting these ecosystems during the summer [68]. The combinatory effects of salinity and low nutrient levels could be related to differentiation between RF and Al locations. Otherwise, PL were considered as part of the physiological adaptations of cell membrane to cope with lower levels of salinities [46], a feature that is worth being investigated in future studies. Meanwhile, these finding highlights that the adaptation of the membrane lipids from seaweeds to environmental conditions can be somewhat unpredictable and strictly depend on different factors. Thus, robust methodologies to address information at molecular level are requested for trace proposes, strain selection or even bioprospection.

## 5. Conclusions

The present study found evidence supporting that site-specific lipid molecular species contributed to the reliable allocation of geographic origin of *Ulva* species. Twenty-five lipid species were highlighted as responding to site-specific conditions. This exploratory analysis showed a remarkable phenotypic plasticity of *Ulva* species lipidome and provided new insights into the relevant impact of environmental conditions on the response of cell membrane lipids. This knowledge encourages further investigation into the lipidome plasticity of seaweeds in general and *Ulva* spp. in particular, namely, the effect of seasonal and interannual shifts in abiotic parameters, of culture conditions when employing land-based approaches, as well as larger scales of geographical variation (e.g., latitudinal clines). The knowledge of natural variation of *Ulva* spp. lipid composition holds potential for traceability purposes and strain selection, contributing towards a safe and quality biomass for wide consumer acceptance.

## Figures and Tables

**Table 1:** Lipid species identified by HILIC–LC–MS in *Ulva* spp. detected at specific geographic origins: Albufeira (Al), Peniche (Pe), Ria Arousa (RAr), Ria de Aveiro (RAv), Ria Formosa (RF), Ria de Pontevedra (RP), Ria de Vigo (RV), Sado Estuary (SE), and Viana do Castelo (VC). Labels of lipid species are according to the notation: AAAA (C:N), (AAAA, lipid class; C, total carbon atoms; N, total double bonds of fatty acid substituents). (D—detected; and ND—not detected).

	Al	Pe	RAr	RAv	RF	RP	RV	SE	VC
**DGMG (16:1)**	D	ND	D	D	D	ND	D	D	ND
**DGMG (16:2)**	ND	ND	ND	ND	ND	ND	D	ND	ND
**DGMG (16:3)**	ND	ND	ND	ND	ND	ND	D	ND	ND
**DGMG (16:4)**	ND	ND	ND	D	ND	ND	ND	ND	ND
**DGDG (30:1)**	ND	ND	D	ND	D	ND	ND	D	D
**DGDG (36:3)**	ND	D	D	D	D	D	D	D	D
DGDG **(38:9)**	D	ND	D	D	D	ND	D	D	D
**SQDG (28:0)**	D	ND	D	D	D	ND	D	ND	D
**SQDG (30:1)**	D	D	D	D	D	ND	D	D	D
**DGTS (28:0)**	D	ND	D	D	D	ND	D	D	D
**DGTS (40:5)**	D	D	D	D	D	D	D	ND	D
**DGTS (42:11)**	D	D	D	ND	D	D	D	D	D
**MGTS (20:0)**	D	D	D	D	D	D	ND	D	D
**PC (30:0)**	D	D	D	D	D	ND	D	D	ND
**PC (34:4)**	D	D	D	D	D	ND	D	D	D
**PC (38:8)**	D	D	D	D	D	ND	D	D	D
**PC (40:10)**	D	D	D	ND	D	ND	D	D	D
**PC (40:7)**	D	D	D	D	D	ND	D	D	D
**LPE (22:5)**	D	D	D	ND	D	D	D	ND	D
**PE (34:5)**	D	D	ND	D	D	D	D	D	ND
**PE (36:6)**	D	D	ND	ND	D	ND	D	D	ND
**PE (40:9)**	D	D	D	ND	D	ND	D	ND	D
**LPG (16:1)**	D	D	D	D	D	D	D	D	ND

**Table 2:** Top 25 lipid species sorted by Tukey’s HSD test displaying the lowest q-values. Lipid molecular species were identified by HILIC-ESI-MS and assigned by MS/MS. MGDG and DGDG were identified by HILIC-ESI-MS as [M + NH_4_]^+^ ions; DGTS, MGTS, PC, PE and LPE were identified as [M + H]^+^ ions; SQDG and LPG were identified as [M − H]^−^ ions. Labels of lipid species are according to the notation: AAAA (C:N), (AAAA, lipid class; C, total carbon atoms; N, total double bonds of fatty acid substituents). (a) Lipid molecular species identification was based on retention time and mass accuracy. (b) Lipid molecular species identification included MS/MS based analysis using diagnostic ions. Asterisked samples refer to specific lipid molecular species represented in Table 1.

*m/z*	Lipid Species (C:N)	Fatty Acyl Chain	Formula
**[M + NH_4_]^+^**			
732.4677	MGDG (32:8)	**16:4-16:4**	C41H66NO10
742.5443	**MGDG (32:3)**	**16:3-16:0 and 18:3-14:0**	C41H76NO10
748.5919	MGDG (32:0)	**16.0-16:0**	C41H82NO10
762.5133	**MGDG (34:7)**	**18:3-16:4**	C43H72NO10
786.5145	**MGDG (36:9)**	**20:5-16:4**	C45H72NO10
960.6582	DGDG (36:3) *	**18:2-18:1**	C51H94O15N
**[M + H]^+^**			
656.5451	DGTS (28:0) *	**14:0-14:0 and 16:0-12:0**	C38H74O7N
808.6058	**DGTS (40:8)**	**22:5-18:3**	C50H82O7N
810.624	DGTS (40:7) *	**22:5-18:2**	C50H84O7N
812.6424	**DGTS (40:6)**	**22:5-18:1**	C50H86O7N
814.6572	DGTS (40:5) *	**22:1-18:4**	C50H88O7N
830.5900	DGTS (42:11) *	**b)**	C52H80O7N
834.6232	**DGTS (42:9)**	**22:5-20:4**	C52H84O7N
860.6368	**DGTS (44:10)**	**22:5-22:5**	C54H86O7N
502.4104	MGTS (18:0)	**18:0**	C28H56O6N
558.4716	MGTS (22:0)	**22:0**	C32H64O6N
706.5372	PC (30:0) *	**a)**	C38H77NO8P
802.53668	PC (38:8)	**b)**	C46H77NO8P
826.5352	PC (40:10) *	**b)**	C48H77NO8P
832.5826	PC (40:7)	**a)**	C48H83NO8P
528.3084	LPE (22:5) *	**22:5**	C27H47NO7P
786.5055	PE (40:9) *	**a)**	C45H73O8NP
**[M − H]^−^**			
737.4507	SQDG (28:0) *	**14:0-14:0 and 12:0-16:0**	C37H69O12S
763.466	SQDG (30:1) *	**16:1-14:0**	C39H71O12S
481.2569	LPG (16:1) *	**16:1**	C22H42O9P

## Supplementary Materials

The following are available online at https://www.mdpi.com/2218-273X/10/3/489/s1, Figure S1: title, Table S1: title, Video S1: title.

## Glossary

Al	Albufeira
DGDG	Digalactosyl diacylglycerol
DGMG	Digalactosyl monoacylglycerol
DGTS	Diacylglyceryl-3-O-4-N,N,N-trimethyl-homoserine
DW	Dry Weight basis
HILIC	Hydrophilic interaction liquid chromatography
LPC	Lyso-phosphatidylcholine
LPE	Lyso-phosphatidylethanolamine
LPG	Lyso-phosphatidylglycerol
LPI	Lyso-phosphatidylinositol
*m/z*	Mass-to-charge ratio
MGDG	Monogalactosyl diacylglycerol
MGMG	Monogalactosyl monoacylglycerol
MGTS	Monocylglyceryl-3-O-4-(N,N,N-trimethyl) homoserine
MS	Mass spectrometry
MS/MS	Tandem mass spectrometry
PC	Phosphatidylcholine
Pe	Peniche
PE	Phosphatidylethanolamine
PG	Phosphatidylglycerol
PI	Phosphatidylinositol
Pi	Inorganic phosphate
PUFA	Polyunsaturated fatty acid
Rar	Ria Arousa
RAv	Ria de Aveiro
RF	Ria Formosa
RP	Ria de Pontevedra
RV	Ria de Vigo
SE	Sado Estuary
SFA	Saturated fatty acid
SQDG	Sulfoquinovosyl diacylglycerol
SQMG	Sulfoquinovosyl monoacylglycerol
VC	Viana do Castelo
TLs	Total lipids
