# Supplementary material for: Site-Specific Lipidomic Signatures of Sea Lettuce (Ulva spp., Chlorophyta) Hold the Potential to Trace Their Geographic Origin
Source: Biomolecules. 2020 Mar 23;10(3):489. doi: 10.3390/biom10030489 (PMC7175330; doi:10.3390/biom10030489)
Supplement: Supplementary file 1 [file biomolecules-10-00489-s001.pdf]

Site-specific lipidomic signatures of sea lettuce (*Ulva* spp., Chlorophyta) hold the potential to trace their geographic origin

Elisabete da Costa [1,2], Fernando Ricardo [1,3], Tânia Melo [1,2], Renato Mamede [3], Maria H. Abreu [4], Pedro Domingues [1,2], M. Rosário Domingues [1,2,] Ricardo Calado [3,*]

Mass Spectrometry Centre, LAQV-REQUIMTE, Department of Chemistry, University of Aveiro, Santiago University Campus, 3810-193 Aveiro, Portugal; elisabetecosta@ua.pt (E.d.C.); taniamelo@ua.pt (T.M.); p.domingues@ua.pt (P.D.); mrd@ua.pt (M.R.D.)

[2] ECOMARE, CESAM, Department of Chemistry, University of Aveiro, Santiago University Campus, 3810-193 Aveiro, Portugal

[3] ECOMARE, CESAM, Department of Biology, University of Aveiro, Santiago University Campus, 3810-193 Aveiro, Portugal; fafr@ua.pt (F.R.); renatomamede@ua.pt (R.M.); rjcalado@ua.pt (R.C.)

[4] ALGAplus- Production and Trading of Seaweed and Derived Products Ltd., 3830-196 Ílhavo, Portugal; helena.abreu@algaplus.pt (M.H.A.)

* Correspondence: rjcalado@ua.pt (R.C.)

Table S1. Biochemical composition of *Ulva* spp. specimens from Albufeira (Al), Peniche (Pe), Ria Arousa (RAr), Ria de Aveiro (RAv), Ria Formosa (RF), Ria de Pontevedra (RP), Ria de Vigo (RV), Sado Estuary (SE), and Viana do Castelo (VC), mean ±S D, % (*n* = 5).

	**Lipid content**		**Protein**		**Carbohydrates and others**		**Ash**	
	Mean, %	±SD, %	Mean, %	±SD, %	Mean, %	±SD, %	Mean, %	±SD, %
Al	**0.51**	0.06	**9.08**	0.41	**71.93**	1.62	**18.48**	1.92
Pe	**1.20**	0.28	**18.13**	1.26	**53.36**	1.30	**27.31**	0.82
RAr	**0.50**	0.12	**6.59**	0.34	**67.49**	1.59	**25.42**	1.57
RAv	**0.40**	0.07	**8.55**	0.29	**63.26**	2.40	**27.79**	2.51
RF	**0.34**	0.06	**6.23**	0.27	**78.67**	0.31	**14.77**	0.09
RP	**1.77**	0.07	**9.90**	0.84	**66.16**	3.95	**22.17**	3.91
RV	**0.47**	0.08	**4.70**	0.31	**69.20**	1.41	**25.63**	1.26
SE	**0.39**	0.13	**12.57**	0.48	**73.56**	1.15	**13.48**	0.80
VC	**0.64**	0.05	**8.60**	0.18	**70.57**	0.53	**20.19**	0.41

Table S2. Biochemical parameters of *Ulva* spp. from nine locations along the Atlantic western and south-western Iberian coast: Albufeira (Al), Ria de Aveiro (RAv), Peniche (Pe), Ria Arousa (RAr), Ria Formosa (RF), Ria de Pontevedra (RP), Ria de Vigo (RV), Sado Estuary (SE), and Viana do Castelo (VC). Significant difference ($p < 0.05$) among different locations are highlighted in grey shaded cells and bold font numbers.

Contrast		Lipids	Protein	Carbohydrates	Ash
Al	RAv	0.5492	0.8439	**<0.0001**	**<0.0001**
	Pe	**<0.0001**	**<0.0001**	**<0.0001**	**<0.0001**
	RAr	1.0000	**<0.0001**	**0.0017**	**<0.0001**
	RF	**0.0254**	**<0.0001**	**0.0001**	**0.0007**
	RP	**<0.0001**	0.9858	**0.0009**	**0.0151**
	RV	0.9989	**<0.0001**	0.1372	**<0.0001**
	SE	0.3542	**<0.0001**	0.9484	**<0.0001**
	VC	0.7103	1.0000	0.8604	0.7508
RAv	Pe	**<0.0001**	**<0.0001**	**<0.0001**	1.0000
	RAr	0.6985	**0.0015**	0.9304	0.7429
	RF	0.8923	**<0.0000**	**<0.0001**	**<0.0001**
	RP	**<0.0001**	**<0.0001**	0.9663	**0.0006**
	RV	0.9258	**<0.0001**	0.1749	0.8323
	SE	1.0000	**<0.0001**	**<0.0001**	**<0.0001**
	VC	**0.0087**	1.0000	**0.0035**	**<0.0001**
Pe	RAr	**<0.0001**	**<0.0001**	**<0.0001**	0.9073
	RF	**<0.0001**	**<0.0001**	**<0.0001**	**<0.0001**
	RP	0.0593	**<0.0001**	**<0.0001**	**0.0024**
	RV	**<0.0001**	**<0.0001**	**<0.0001**	0.9529
	SE	**<0.0001**	**<0.0001**	**<0.0001**	**0.0001**
	VC	**<0.0001**	**<0.0001**	**<0.0001**	**<0.0001**
RAr	RF	**0.0477**	0.2034	**<0.0001**	**<0.0001**
	RP	**0.0001**	**<0.0001**	1.0000	0.1828
	RV	0.9999	**<0.0001**	0.9243	1.0000
	SE	0.4965	**<0.0001**	**<0.0001**	**<0.0001**
	VC	0.5618	**<0.0001**	0.2018	**0.0004**
RF	RP	**<0.0001**	**<0.0001**	**<0.0001**	**<0.0001**
	RV	0.1520	**<0.0001**	**<0.0001**	**<0.0001**
	SE	0.9716	**<0.0001**	**0.0166**	0.7159
	VC	**0.0001**	**<0.0001**	**<0.0001**	**<0.0001**
RP	RV	**<0.0001**	**<0.0001**	0.8661	0.1259
	SE	**<0.0001**	**<0.0001**	**<0.0001**	**<0.0001**
	VC	**<0.0001**	0.9970	0.1415	0.6842
RV	SE	0.7969	**<0.0001**	**0.0024**	**<0.0001**
	VC	0.2734	**<0.0001**	0.9475	**0.0002**
SE	VC	**0.0032**	**<0.0001**	0.1384	**<0.0001**

Table S3. Glycolipids, phospholipids, and betaine lipids identified by LC–MS and MS/MS on *Ulva* spp. specimens collected from nine different locations (mass error < 5 ppm). Observed *m/z* and respective error were checked for all samples. Numbers in parenthesis (C:N) indicates the total number of carbon atoms (C) and total double bonds (N) in the fatty acyl chains.

Lipid species (C:N)	Calculated *m/z*	Observed *m/z*	Error (ppm)	Formula
**$[M + NH_4]^+$**				
MGMG (16:4)	502.3016	502.3005	**−2.2078**	C25H44NO9
MGMG (16:3)	504.3173	504.3175	**0.4779**	C25H46NO9
MGMG (16:2)	506.3329	506.3317	**−2.3878**	C25H48NO9
MGMG (16:1)	508.3486	508.3475	**−2.0832**	C25H50NO9
MGMG (16:0)	510.3642	510.3630	**−2.3513**	C25H52NO9
MGMG (18:4)	530.3329	530.3320	**−1.7140**	C27H48NO9
MGMG (18:3)	532.3486	532.3482	**−0.6744**	C27H50NO9
MGMG (18:1)	536.3799	536.3784	**−2.7201**	C27H54NO9
DGMG (16:4)	664.3544	664.3524	**−3.0616**	C31H54NO14
DGMG (16:3)	666.3701	666.3692	**−1.3266**	C31H56NO14
DGMG (16:2)	668.3857	668.3846	**−1.6966**	C31H58NO14
DGMG (16:1)	670.4013	670.4006	**−1.1694**	C31H60NO14
DGMG (16:0)	672.4170	672.4192	**3.2718**	C31H62NO14
MGDG (30:4)	712.5000	712.4971	**−4.0702**	C39H70NO10
MGDG (32:8)	732.4687	732.4677	**−1.3652**	C41H66NO10
MGDG (32:7)	734.4843	734.4825	**−2.4507**	C41H68NO10
MGDG (32:6)	736.4994	736.4983	**−1.4936**	C41H70NO10
MGDG (32:5)	738.5156	738.5149	**−0.9803**	C41H72NO10
MGDG (32:4)	740.5307	740.5295	**−1.6205**	C41H74NO10
MGDG (32:3)	742.5464	742.5443	**−2.8281**	C41H76NO10
MGDG (32:2)	744.5626	744.5604	**−2.9548**	C41H78NO10
MGDG (32:1)	746.5777	746.5767	**−1.3394**	C41H80NO10
MGDG (32:0)	748.5939	748.5919	**−2.6369**	C41H82NO10
MGDG (34:8)	760.5000	760.4995	**−0.6575**	C43H70NO10
MGDG (34:7)	762.5156	762.5133	**−3.0478**	C43H72NO10
MGDG (34:6)	764.5312	764.5306	**−0.8803**	C43H74NO10
MGDG (34:5)	766.5470	766.5461	**−1.0750**	C43H76NO10
MGDG (34:4)	768.5626	768.5610	**−2.0818**	C43H78NO10
MGDG (34:3)	770.5782	770.5767	**−1.9777**	C43H80NO10
MGDG (34:2)	772.5933	772.5911	**−2.8476**	C43H82NO10
MGDG (34:1)	774.6090	774.6075	**−1.9365**	C43H84NO10
MGDG (36:9)	786.5156	786.5145	**−1.3986**	C45H72NO10
MGDG (36:8)	788.5313	788.5295	**−2.2447**	C45H74NO10
MGDG (36:7)	790.5469	790.5463	**−0.7590**	C45H76NO10

MGDG (36:6)	792.5625	792.5629	**0.5047**	C45H78NO10
MGDG (36:5)	794.5782	794.5774	**−1.0068**	C45H80NO10
MGDG (36:4)	796.5933	796.5912	**−2.6362**	C45H82NO10
MGDG (38:9)	814.5469	814.5468	**−0.1522**	C47H76NO10
DGDG (28:0)	854.5841	854.5838	**−0.3510**	C43H84O15N
DGDG (30:1)	880.5997	880.5990	**−0.7949**	C45H86O15N
DGDG (32:4)	902.5841	902.5827	**−1.5511**	C47H84O15N
DGDG (32:3)	904.5997	904.5982	**−1.6582**	C47H86O15N
DGDG (32:2)	906.6154	906.6137	**−1.8751**	C47H88O15N
DGDG (32:1)	908.6310	908.6281	**−3.1916**	C47H90O15N
DGDG (32:0)	910.6467	910.6448	**−2.0864**	C47H92O15N
DGDG (34:8)	922.5528	922.5513	**−1.6259**	C49H80O15N
DGDG (34:7)	924.5684	924.5654	**−3.2448**	C49H82O15N
DGDG (34:6)	926.5841	926.5827	**−1.5109**	C49H84O15N
DGDG (34:5)	928.5997	928.5984	**−1.4000**	C49H86O15N
DGDG (34:4)	930.6154	930.6150	**−0.4298**	C49H88O15N
DGDG (34:3)	932.6310	932.6305	**−0.5361**	C49H90O15N
DGDG (34:2)	934.6467	934.6451	**−1.7119**	C49H92O15N
DGDG (34:1)	936.6623	936.6592	**−3.3096**	C49H94O15N
DGDG (36:7)	952.5997	952.5989	**−0.8398**	C51H86O15N
DGDG (36:6)	954.6154	954.6139	**−1.5713**	C51H88O15N
DGDG (36:5)	956.6310	956.6279	**−3.2405**	C51H90O15N
DGDG (36:4)	958.6467	958.6437	**−3.1294**	C51H92O15N
DGDG (36:3)	960.6623	960.6582	**−4.2679**	C51H94O15N
DGDG (36:2)	962.6780	962.6746	**−3.5318**	C51H96O15N
DGDG (38:9)	976.5997	976.5982	**−1.5359**	C53H86O15N
**$[M + H]^+$**				
DGTS (28:0)	656.5465	656.5451	**−2.1324**	C38H74O7N
DGTS (30:2)	680.5465	680.5440	**−3.6735**	C40H74O7N
DGTS (30:1)	682.5622	682.5608	**−2.0511**	C40H76O7N
DGTS (30:0)	684.5778	684.5761	**−2.4833**	C40H78O7N
DGTS (32:4)	704.5465	704.5448	**−2.4129**	C42H74O7N
DGTS (32:3)	706.5622	706.5601	**−2.9721**	C42H76O7N
DGTS (32:2)	708.5778	708.5762	**−2.2580**	C42H78O7N
DGTS (32:1)	710.5935	710.5917	**−2.5331**	C42H80O7N
DGTS (32:0)	712.6091	712.6056	**−4.9115**	C42H82O7N
DGTS (34:8)	724.5152	724.5144	**−1.1442**	C44H70O7N
DGTS (34:7)	726.5309	726.5282	**−3.6874**	C44H72O7N
DGTS (34:6)	728.5465	728.5447	**−2.4707**	C44H74O7N
DGTS (34:5)	730.5622	730.5610	**−1.6426**	C44H76O7N
DGTS (34:4)	732.5778	732.5761	**−2.3206**	C44H78O7N
DGTS (34:3)	734.5935	734.5913	**−2.9949**	C44H80O7N
DGTS (34:2)	736.6091	736.6067	**−3.2582**	C44H82O7N

DGTS (34:1)	738.6248	738.6216	**−4.3324**	C44H84O7N
DGTS (36:8)	752.5465	752.5447	**−2.4185**	C46H74O7N
DGTS (36:7)	754.5622	754.5591	**−4.1083**	C46H76O7N
DGTS (36:6)	756.5778	756.5747	**−4.0974**	C46H78O7N
DGTS (36:5)	758.5935	758.5918	**−2.2410**	C46H80O7N
DGTS (36:4)	760.6091	760.6068	**−3.0239**	C46H82O7N
DGTS (36:3)	762.6248	762.6238	**−1.3113**	C46H84O7N
DGTS (36:2)	764.6404	764.6398	**−0.7847**	C46H86O7N
DGTS (38:10)	776.5465	776.5430	**−4.5445**	C48H74O7N
DGTS (38:9)	778.5622	778.5593	**−3.7248**	C48H76O7N
DGTS (38:8)	780.5778	780.5750	**−3.5871**	C48H78O7N
DGTS (38:7)	782.5935	782.5902	**−4.2167**	C48H80O7N
DGTS (38:6)	784.6091	784.6086	**−0.6373**	C48H82O7N
DGTS (38:5)	786.6248	786.6233	**−1.9069**	C48H84O7N
DGTS (38:4)	788.6404	788.6377	**−3.4236**	C48H86O7N
DGTS (40:9)	806.5935	806.5911	**−2.9755**	C50H80O7N
DGTS (40:8)	808.6091	808.6058	**−4.0811**	C50H82O7N
DGTS (40:7)	810.6248	810.6240	**−0.9869**	C50H84O7N
DGTS (40:6)	812.6404	812.6424	**2.4611**	C50H86O7N
DGTS (40:5)	814.6561	814.6572	**1.3503**	C50H88O7N
DGTS (40:4)	816.6717	816.6706	**−1.3469**	C50H90O7N
DGTS (42:11)	830.5935	830.5900	**−4.1886**	C52H80O7N
DGTS (42:10)	832.6091	832.6056	**−4.2373**	C52H82O7N
DGTS (42:9)	834.6248	834.6232	**−1.8919**	C52H84O7N
DGTS (44:10)	860.6404	860.6368	**−4.2166**	C54H86O7N
MGTS (14:0)	446.3482	446.3473	**−1.9357**	C24H48O6N
MGTS (16:4)	466.3169	466.3154	**−3.1395**	C26H44O6N
MGTS (16:3)	468.3325	468.3311	**−3.0192**	C26H46O6N
MGTS (16:2)	470.3482	470.3477	**−0.9865**	C26H48O6N
MGTS (16:1)	472.3638	472.3623	**−3.2052**	C26H50O6N
MGTS (16:0)	474.3795	474.3786	**−1.8972**	C26H52O6N
MGTS (18:4)	494.3482	494.3468	**−2.7592**	C28H48O6N
MGTS (18:3)	496.3638	496.3628	**−2.0429**	C28H50O6N
MGTS (18:2)	498.3794	498.3791	**−0.7304**	C28H52O6N
MGTS (18:1)	500.3951	500.3948	**−0.6275**	C28H54O6N
MGTS (18:0)	502.4108	502.4104	**−0.7245**	C28H56O6N
MGTS (20:5)	520.3638	520.3624	**−2.7173**	C30H50O6N
MGTS (20:4)	522.3795	522.3784	**−2.0368**	C30H52O6N
MGTS (20:0)	530.4421	530.4403	**−3.3255**	C30H60O6N
MGTS (22:5)	548.3951	548.3941	**−1.8490**	C32H54O6N
MGTS (22:1)	556.4577	556.4561	**−2.9005**	C32H62O6N
MGTS (22:0)	558.4734	558.4716	**−3.1586**	C32H64O6N
PC (30:3)	700.4917	700.4884	**−4.7567**	C38H71NO8P

PC (30:0)	706.5387	706.5372	**−2.0975**	C38H77NO8P
PC (32:3)	728.5230	728.5227	**−0.4557**	C40H75NO8P
PC (32:2)	730.5387	730.5381	**−0.7967**	C40H77NO8P
PC (32:0)	734.5700	734.5668	**−4.3318**	C40H81NO8P
PC (34:5)	752.5230	752.5220	**−1.3714**	C42H75NO8P
PC (34:4)	754.5387	754.5355	**−4.2171**	C42H77NO8P
PC (34:3)	756.5543	756.5531	**−1.6271**	C42H79NO8P
PC (34:2)	758.5700	758.5683	**−2.2173**	C42H81NO8P
PC (34:1)	760.5856	760.5830	**−3.4605**	C42H83NO8P
PC (36:6)	778.5387	778.5371	**−2.0320**	C44H77NO8P
PC (36:5)	780.5543	780.5529	**−1.8333**	C44H79NO8P
PC (36:4)	782.5700	782.5674	**−3.2994**	C44H81NO8P
PC (36:3)	784.5856	784.5840	**−2.0801**	C44H83NO8P
PC (36:2)	786.6013	786.5982	**−3.9181**	C44H85NO8P
PC (38:8)	802.5387	802.5367	**−2.4946**	C46H77NO8P
PC (38:7)	804.5543	804.5526	**−2.1515**	C46H79NO8P
PC (38:6)	806.5700	806.5692	**−0.9695**	C46H81NO8P
PC (38:5)	808.5856	808.5821	**−4.3681**	C46H83NO8P
PC (38:4)	810.6013	810.5975	**−4.6657**	C46H85NO8P
PC (40:10)	826.5387	826.5352	**−4.2127**	C48H77NO8P
PC (40:9)	828.5543	828.5507	**−4.3823**	C48H79NO8P
PC (40:8)	830.5699	830.5671	**−3.4699**	C48H81NO8P
PC (40:7)	832.5856	832.5826	**−3.6417**	C48H83NO8P
LPE (20:4)	502.2934	502.2914	**−3.9160**	C25H45NO7P
LPE (22:5)	528.3090	528.3084	**−1.1679**	C27H47NO7P
PE (30:1)	662.4761	662.4748	**−1.9352**	C35H69NO8P
PE (30:0)	664.4917	664.4894	**−3.5094**	C35H71NO8P
PE (32:4)	684.4604	684.4576	**−4.1376**	C37H67NO8P
PE (32:2)	688.4917	688.4908	**−1.3537**	C37H71O8NP
PE (32:1)	690.5074	690.5051	**−3.3048**	C37H73NO8P
PE (32:0)	692.5230	692.5201	**−4.2338**	C37H75NO8P
PE (34:5)	710.4761	710.4737	**−3.3527**	C39H69NO8P
PE (34:3)	714.5074	714.5061	**−1.7942**	C39H73O8NP
PE (34:2)	716.5230	716.5216	**−1.9985**	C39H75NO8P
PE (34:1)	718.5387	718.5355	**−4.4284**	C39H77NO8P
PE (36:6)	736.4917	736.4916	**−0.1792**	C41H71NO8P
PE (36:5)	738.5074	738.5058	**−2.1422**	C41H73O8NP
PE (36:4)	740.5230	740.5209	**−2.8790**	C41H75NO8P
PE (36:3)	742.5387	742.5364	**−3.0732**	C41H77NO8P
PE (36:2)	744.5543	744.5515	**−3.8023**	C41H79O8NP
PE (40:9)	786.5074	786.5055	**−2.3929**	C45H73O8NP
PE (40:8)	788.5230	788.5235	**0.5935**	C45H75O8NP
LPC (16:0)	496.3403	496.3395	**−1.6440**	C24H51NO7P

LPC (18:1)	522.3560	522.3553	**−1.2635**	C26H51NO7P
LPC (22:6)	568.3403	568.3395	**−1.4252**	C30H51NO7P
**[M − H]⁻**				
SQDG (28:0)	737.4510	737.4507	**−0.3756**	C37H69O12S
SQDG (30:1)	763.4666	763.4660	**−0.8213**	C39H71O12S
SQDG (30:0)	765.4823	765.4819	**−0.4925**	C39H73O12S
SQDG (32:4)	785.4510	785.4497	**−1.6258**	C41H69O12S
SQDG (32:3)	787.4666	787.4657	**−1.1772**	C41H71O12S
SQDG (32:2)	789.4822	789.4785	**−4.7841**	C41H73O12S
SQDG (32:1)	791.4979	791.4969	**−1.2975**	C41H75O12S
SQDG (32:0)	793.5136	793.5125	**−1.3560**	C41H77O12S
SQDG (33:1)	805.5136	805.5128	**−0.9634**	C42H77O12S
SQDG (34:4)	813.4823	813.4829	**0.7658**	C43H73O12S
SQDG (34:3)	815.4979	815.4973	**−0.7689**	C43H75O12S
SQDG (34:2)	817.5136	817.5110	**−3.1510**	C43H77O12S
SQDG (34:1)	819.5292	819.5280	**−1.4972**	C43H79O12S
SQDG (36:5)	839.4979	839.4967	**−1.4616**	C45H75O12S
SQDG (36:4)	841.5136	841.5127	**−1.0410**	C45H77O12S
SQDG (36:3)	843.5292	843.5257	**−4.1812**	C45H79O12S
SQDG (36:2)	845.5449	845.5442	**−0.8007**	C45H81O12S
SQDG (36:1)	847.5605	847.5593	**−1.4477**	C45H83O12S
SQMG (16:0)	555.2839	555.2833	**−1.1021**	C25H47O11S
PI (34:2)	833.5180	833.5156	**−2.8794**	C43H78O13P
PI (38:10)	831.5024	831.4987	**−4.4498**	C47H70O13P
PI (34:3)	873.4554	873.4547	**−0.8014**	C43H76O13P
PI (38:4)	885.5493	885.5516	**2.5973**	C47H82O13P
PI (38:8)	877.4867	877.4866	**−0.1140**	C47H74O13P
PI (42:11)	927.5024	927.5010	**−1.5094**	C51H76O13P
PG (36:2)	773.5333	773.5322	**−1.4220**	C42H78O10P
PG (36:5)	767.4863	767.4858	**−0.6515**	C42H72O10P
PG (36:3)	771.5176	771.5172	**−0.5185**	C42H76O10P
PG (40:6)	821.5333	821.5329	**−0.4869**	C46H78O10P
PG (34:4)	741.4707	741.4685	**−2.9671**	C40H70O10P
PG (34:3)	743.4863	743.4851	**−1.6140**	C40H72O10P
PG (34:2)	745.5020	745.5011	**−1.2072**	C40H74O10P
PG (34:1)	747.5176	747.5161	**−2.0066**	C40H76O10P
PG (32:2)	717.4707	717.4719	**1.6725**	C38H70O10P
PG (32:1)	719.4863	719.4851	**−1.6679**	C38H72O10P
PG (32:0)	721.5020	721.5013	**−0.9702**	C38H74O10P
LPG (16:1)	481.2566	481.2569	**0.5236**	C22H42O9P

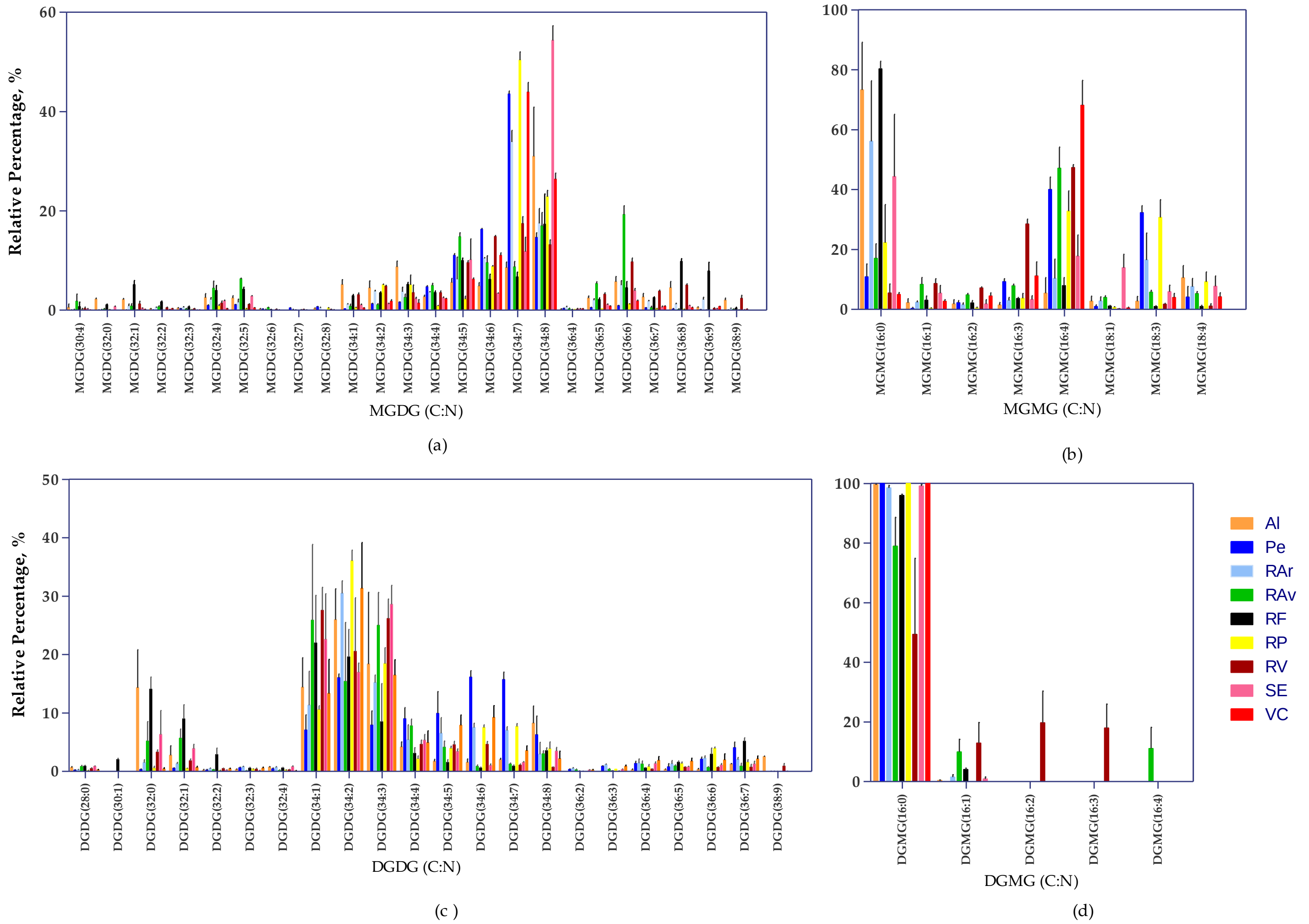

Figure S1. Relative percentage (%) of galactolipids identified by HILIC-LC-MS of *Ulva* spp. specimens from different geographic origins: a) MGDG, b) MGMG, c) DGDG, d) DGMG. Numbers in parentheses (C:N) indicates the number of total carbon atoms: total double bonds of fatty acyl chains.

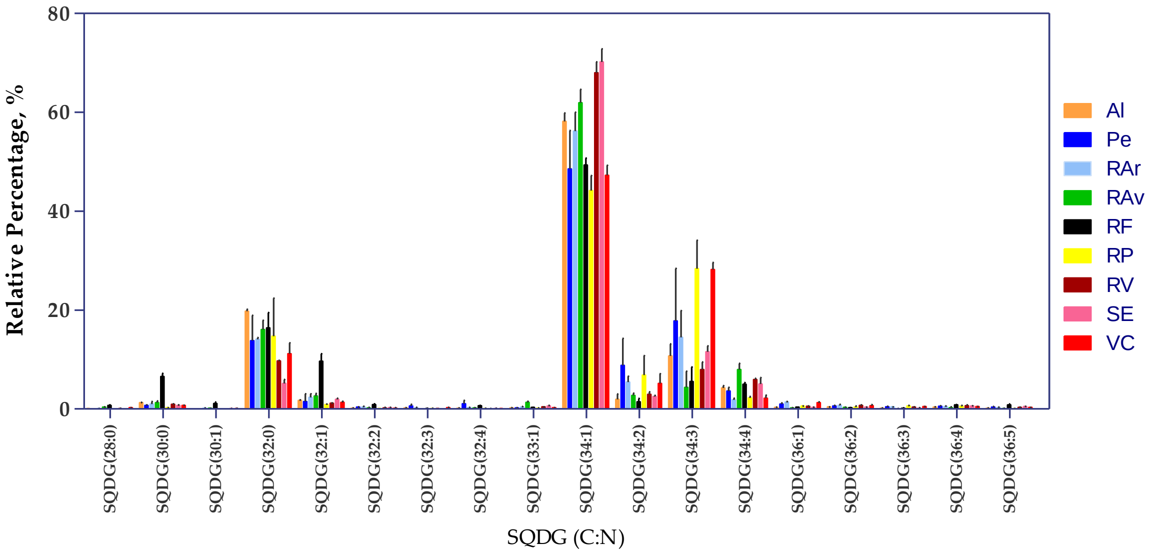

Figure S2. Relative percentage (%) of sulfolipids identified by HILIC-LC-MS of *Ulva* spp. specimens from different geographic origins: SQDG. Numbers in parentheses (C:N) indicates the number of total carbon atoms: total double bonds of fatty acyl chains.

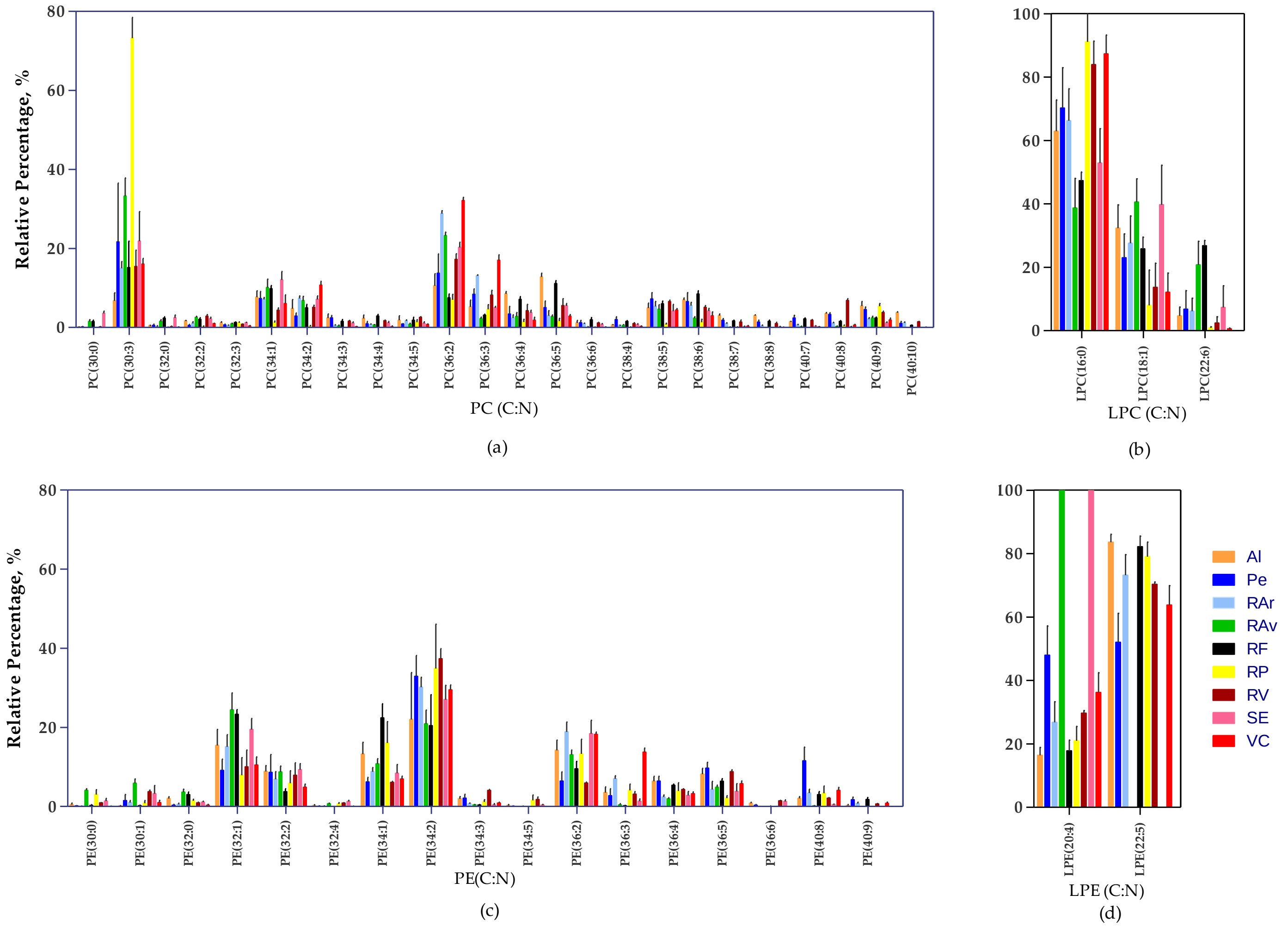

Figure S3. Relative percentage (%) of phospholipids identified by HILIC-LC-MS of *Ulva* spp. specimens from different geographic origins: a) PC, b) LPC, c) PE, d) LPE. Numbers in parentheses (C:N) indicates the number of total carbon atoms: total double bonds of fatty acyl chains.

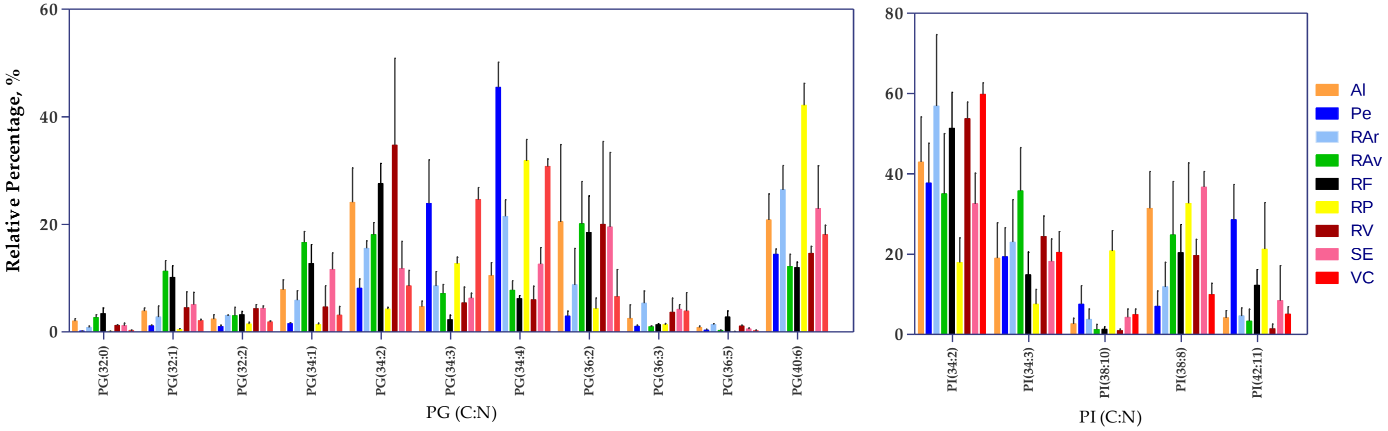

(a) (b)

Figure S4. Relative percentage (%) of phospholipids identified by HILIC-LC-MS of *Ulva* spp. specimens from different geographic origins: a) PG, b) PI. Numbers in parentheses (C:N) indicates the number of total carbon atoms: total double bonds of fatty acyl chains.

.

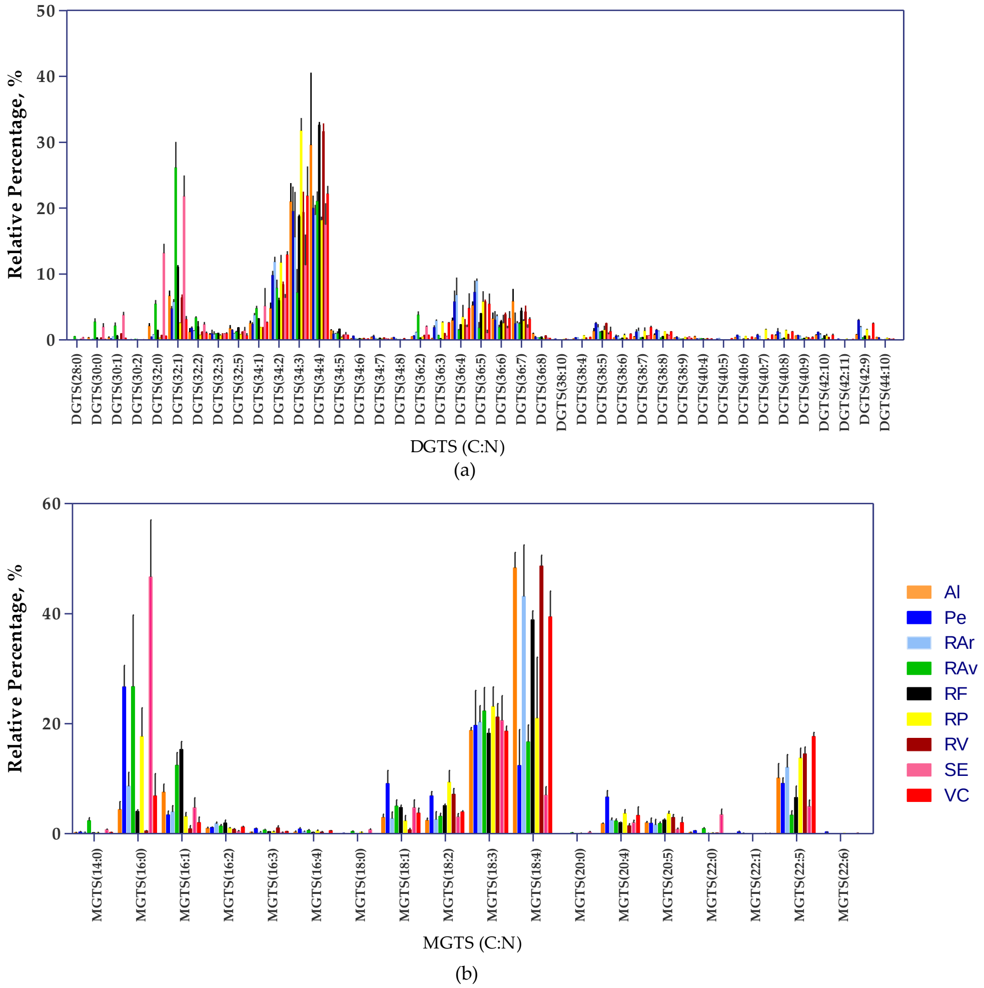

Figure S5. Relative percentage (%) of betaine lipids identified by HILIC-LC-MS of *Ulva* spp. specimens from different geographic origins: a) DGTS, b) MGTS. Numbers in parentheses (C:N) indicates the number of total carbon atoms: total double bonds of fatty acyl chains.

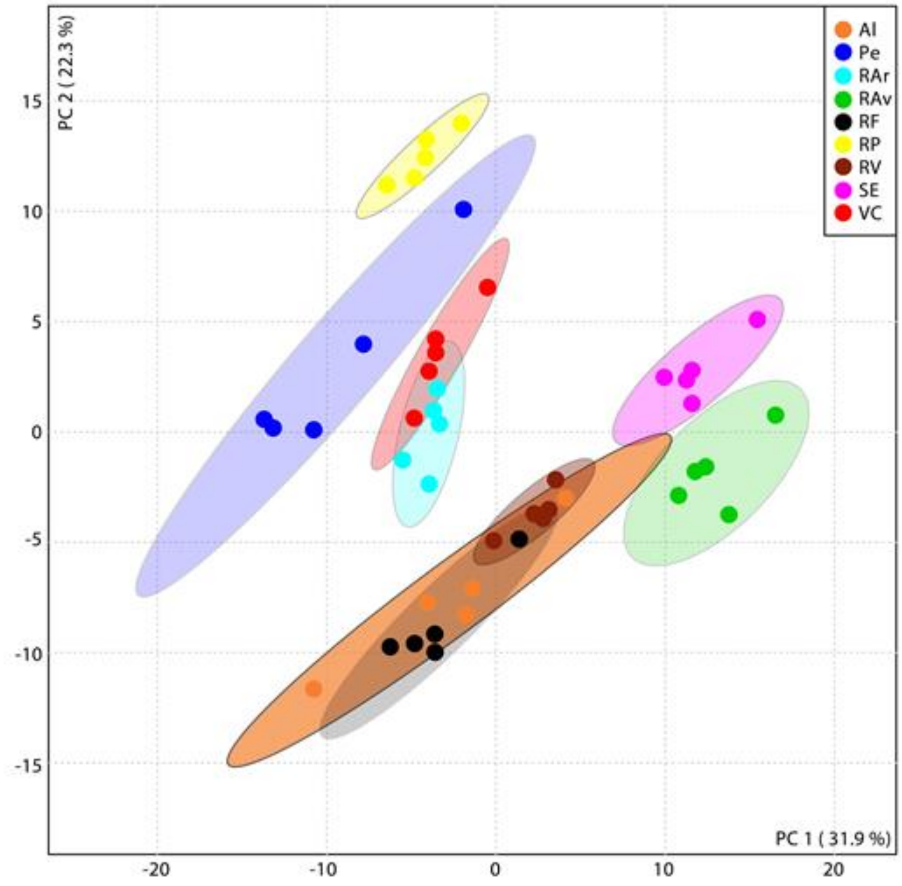

Figure S6. Principal components analysis (PCA) scores plot of two first PCs (PC2 versus PC1) performed on the entire sample of standardized and log-transformed lipid data set acquired by HILIC-LC-MS of the nine geographic origins: Albufeira (Al), Peniche (Pe), Ria Arousa (RA), Ria de Aveiro (RAv), Ria Formosa (RF), Ria Pontevedra (RP), Ria de Vigo (RV), Sado Estuary (SE), and Viana do Castelo (VC).

Table S4. One-way ANOVA (ANalysis Of VAriance) glog transformed and autoscaled HILIC-MS data, followed by post-hoc Tukey's honestly significant difference multiple comparison test and *p*-values correction for multiple testing using Benjamini–Hochberg false discovery rate (FDR, q values) *Ulva* spp. was collected from the nine geographic origins along the Atlantic coast: Albufeira (Al), Ria de Aveiro (RAv), Peniche (Pe), Ria Arousa (RAr), Ria Formosa (RF), Ria de Pontevedra (RP), Ria de Vigo (RV), Sado Estuary (SE), and Viana do Castelo (VC).

Lipid species	FDR	Tukey's HSD
DGTS (28:0)	6.16E-29	RAv-Al; Pe-Al; RF-Al; RP-Al; SE-Al; Pe-RAv; RAr-RAv; RP-RAv; VC-RAv; RAr-Pe; RF-Pe; RV-Pe; SE-Pe; VC-Pe; RP-RAr; SE-RAr; VC-RAr; RP-RF; VC-RF; RV-RP; SE-RP; VC-RP; VC-RV; VC-SE
PC (40:10)	8.90E-26	RAv-Al; Pe-Al; RAr-Al; RF-Al; RP-Al; RV-Al; SE-Al; VC-Al; Pe-RAv; RAr-RAv; RF-RAv; RV-RAv; SE-RAv; VC-RAv; RP-Pe; SE-Pe; VC-Pe; RP-RAr; SE-RAr; VC-RAr; RP-RF; SE-RF; VC-RF; RV-RP; SE-RP; VC-RP; SE-RV; VC-RV
SQDG (28:0)	3.34E-25	Pe-Al; RF-Al; RP-Al; RV-Al; SE-Al; Pe-RAv; RAr-RAv; RP-RAv; RV-RAv; SE-RAv; RAr-Pe; RF-Pe; RV-Pe; VC-Pe; RF-RAr; RP-RAr; SE-RAr; RP-RF; RV-RF; SE-RF; RV-RP; VC-RP; SE-RV; VC-RV; VC-SE
DGDG (36:3)	1.49E-24	RAv-Al; Pe-Al; RAr-Al; RF-Al; RP-Al; RV-Al; SE-Al; VC-Al; Pe-RAv; RAr-RAv; RP-RAv; RV-RAv; VC-RAv; RF-Pe; RP-Pe; RV-Pe; SE-Pe; RF-RAr; RV-RAr; SE-RAr; RP-RF; RV-RF; VC-RF; RV-RP; SE-RV; VC-RV; VC-SE
DGTS (40:5)	4.15E-24	RAv-Al; Pe-Al; RAr-Al; RP-Al; SE-Al; VC-Al; Pe-RAv; RAr-RAv; RF-RAv; RP-RAv; RV-RAv; SE-RAv; VC-RAv; RF-Pe; RP-Pe; RV-Pe; SE-Pe; RF-RAr; RP-RAr; RV-RAr; SE-RAr; RP-RF; SE-RF; VC-RF; RV-RP; SE-RP; VC-RP; SE-RV; VC-RV; VC-SE
DGTS (40:7)	6.55E-23	RAv-Al; Pe-Al; RF-Al; RP-Al; SE-Al; Pe-RAv; RAr-RAv; RF-RAv; RP-RAv; RV-RAv; SE-RAv; VC-RAv; RF-Pe; RV-Pe; SE-Pe; RF-RAr; RP-RAr; RV-RAr; SE-RAr; RP-RF; RV-RF; SE-RF; VC-RF; RV-RP; SE-RP; SE-RV; VC-RV; VC-SE
LPE (22:5)	6.55E-23	RAv-Al; Pe-Al; RAr-Al; SE-Al; VC-Al; Pe-RAv; RAr-RAv; RF-RAv; RP-RAv; RV-RAv; VC-RAv; RF-Pe; RV-Pe; SE-Pe; RV-RAr; SE-RAr; SE-RF; VC-RF; RV-RP; SE-RP; SE-RV; VC-RV; VC-SE
PE (40:9)	6.84E-23	RAv-Al; RF-Al; RP-Al; RV-Al; SE-Al; Pe-RAv; RAr-RAv; RF-RAv; RV-RAv; VC-RAv; RP-Pe; RV-Pe; SE-Pe; RP-RAr; RV-RAr; SE-RAr; RP-RF; SE-RF; RV-RP; VC-RP; SE-RV; VC-SE
DGTS (42:11)	1.42E-21	RAv-Al; Pe-Al; RAr-Al; RF-Al; RP-Al; SE-Al; VC-Al; Pe-RAv; RAr-RAv; RF-RAv; RP-RAv; RV-RAv; SE-RAv; VC-RAv; RF-Pe; RV-Pe; SE-Pe; RF-RAr; RV-RAr; SE-RAr; RP-RF; RV-RF; VC-RF; RV-RP; SE-RP; SE-RV; VC-RV; VC-SE
PC (40:7)	1.97E-20	RAv-Al; RP-Al; SE-Al; VC-Al; Pe-RAv; RAr-RAv; RF-RAv; RP-RAv; RV-RAv; RP-Pe; SE-Pe; VC-Pe; RP-RAr; RP-RF; SE-RF; VC-RF; RV-RP; SE-RP; VC-RP; SE-RV; VC-RV
PC (30:0)	8.55E-20	RAv-Al; RAr-Al; RF-Al; RP-Al; SE-Al; VC-Al; Pe-RAv; RAr-RAv; RP-RAv; RV-RAv; VC-RAv; RF-Pe; RP-Pe; SE-Pe; VC-Pe; RF-RAr; RP-RAr; SE-RAr; VC-RAr; RP-RF; RV-RF; VC-RF; RV-RP; SE-RP; SE-RV; VC-RV; VC-SE
DGTS (42:9)	3.52E-19	RAv-Al; Pe-Al; RAr-Al; RP-Al; SE-Al; VC-Al; Pe-RAv; RAr-RAv; RF-RAv; RP-RAv; RV-RAv; VC-RAv; RF-Pe; RV-Pe; SE-Pe; RF-RAr; RV-RAr; SE-RAr; RP-RF; SE-RF; VC-RF; RV-RP; SE-RP; SE-RV; VC-RV; VC-SE
MGDG (36:9)	6.22E-19	RAv-Al; RF-Al; RP-Al; RV-Al; SE-Al; Pe-RAv; RAr-RAv; RF-RAv; RP-RAv; RV-RAv; SE-RAv; VC-RAv; RF-Pe; RP-Pe; RV-Pe; SE-Pe; RF-RAr; RP-RAr; RV-RAr; SE-RAr; RP-RF; RV-RF; SE-RF; VC-RF; RV-RP; SE-RP; VC-RP; VC-RV; VC-SE
SQDG (30:1)	2.43E-18	RF-Al; RP-Al; RV-Al; Pe-RAv; RF-RAv; RP-RAv; RV-RAv; SE-RAv; RAr-Pe; RF-Pe; RP-Pe; VC-Pe; RF-RAr; RP-RAr; RV-RAr; SE-RAr; RP-RF; RV-RF; SE-RF; VC-RF; RV-RP; SE-RP; VC-RP; VC-RV; VC-SE
MGDG (32:8)	2.84E-18	RAv-Al; Pe-Al; RF-Al; RP-Al; RV-Al; SE-Al; VC-Al; Pe-RAv; RAr-RAv; RF-RAv; RP-RAv; RV-RAv; SE-RAv; RAr-Pe; RF-Pe; RV-Pe; SE-Pe; VC-Pe; RF-RAr; RV-RAr; SE-RAr; VC-RAr; RP-RF; RV-RP; SE-RP; VC-RP
MGDG (34:7)	4.69E-18	RAv-Al; Pe-Al; RAr-Al; RP-Al; VC-Al; Pe-RAv; RAr-RAv; RF-RAv; RP-RAv; RV-RAv; SE-RAv; VC-RAv; RAr-Pe; RF-Pe; RV-Pe; SE-Pe; VC-Pe; RF-RAr; RP-RAr; RV-RAr; SE-RAr; VC-RAr; RP-RF; VC-RF; RV-RP; SE-RP; VC-RV; VC-SE
MGTS (22:0)	5.16E-18	Pe-Al; RAr-Al; RF-Al; RP-Al; RV-Al; VC-Al; RAr-RAv; RF-RAv; RP-RAv; SE-RAv; VC-

		RAv; RAr-Pe; SE-Pe; VC-Pe; RF-RAr; RP-RAr; RV-RAr; SE-RAr; SE-RF; VC-RF; SE-RP; VC-RP; SE-RV; VC-RV; VC-SE
DGTS (40:6)	5.16E-18	RAv-Al; SE-Al; Pe-RAv; RAr-RAv; RF-RAv; RP-RAv; RV-RAv; VC-RAv; RF-Pe; RV-Pe; SE-Pe; RV-RAr; SE-RAr; RP-RF; SE-RF; RV-RP; SE-RP; SE-RV; VC-RV; VC-SE
MGTS (18:0)	6.95E-18	Pe-Al; RAr-Al; RP-Al; RV-Al; SE-Al; VC-Al; Pe-RAv; RAr-RAv; RF-RAv; RV-RAv; VC-RAv; RF-Pe; RP-Pe; SE-Pe; RF-RAr; RP-RAr; SE-RAr; RP-RF; RV-RF; SE-RF; VC-RF; RV-RP; VC-RP; SE-RV; VC-SE
DGTS (40:8)	9.68E-18	RAv-Al; Pe-Al; RP-Al; SE-Al; Pe-RAv; RAr-RAv; RF-RAv; RP-RAv; RV-RAv; VC-RAv; RF-Pe; RV-Pe; SE-Pe; RF-RAr; SE-RAr; RP-RF; SE-RF; VC-RF; RV-RP; SE-RP; SE-RV; VC-RV; VC-SE
DGTS (44:10)	1.56E-17	RAv-Al; RF-Al; RV-Al; SE-Al; Pe-RAv; RAr-RAv; RF-RAv; RP-RAv; RV-RAv; SE-RAv; VC-RAv; RAr-Pe; RF-Pe; RV-Pe; SE-Pe; VC-Pe; RP-RAr; SE-RAr; RP-RF; SE-RF; RV-RP; SE-RP; SE-RV; VC-SE
PC (38:8)	3.13E-17	RAv-Al; RAr-Al; RP-Al; SE-Al; VC-Al; Pe-RAv; RAr-RAv; RF-RAv; RP-RAv; RV-RAv; SE-RAv; RP-Pe; SE-Pe; VC-Pe; RP-RAr; RP-RF; SE-RF; VC-RF; RV-RP; SE-RP; VC-RP; SE-RV; VC-RV
LPG (16:1)	3.77E-17	Pe-Al; SE-Al; VC-Al; Pe-RAv; SE-RAv; VC-RAv; RF-Pe; RP-Pe; SE-Pe; VC-Pe; SE-RAr; VC-RAr; SE-RF; VC-RF; SE-RP; VC-RP; SE-RV; VC-RV
MGDG (32:0)	4.14E-17	RAv-Al; Pe-Al; RAr-Al; RP-Al; RV-Al; SE-Al; VC-Al; Pe-RAv; RF-RAv; RP-RAv; RV-RAv; SE-RAv; VC-RAv; RAr-Pe; RF-Pe; RV-Pe; VC-Pe; RF-RAr; RP-RAr; RV-RAr; SE-RAr; VC-RAr; RP-RF; RV-RF; SE-RF; VC-RF; RV-RP; VC-RP; SE-RV; VC-SE
MGDG (32:3)	1.16E-16	RAv-Al; Pe-Al; SE-Al; VC-Al; Pe-RAv; RAr-RAv; RF-RAv; RP-RAv; SE-RAv; RAr-Pe; RP-Pe; RV-Pe; SE-Pe; VC-Pe; SE-RAr; VC-RAr; RP-RF; RV-RF; SE-RF; VC-RF; SE-RP; SE-RV; VC-SE
DGDG (34:7)	1.50E-16	RAv-Al; Pe-Al; RAr-Al; RP-Al; RV-Al; Pe-RAv; RAr-RAv; RF-RAv; RP-RAv; SE-RAv; VC-RAv; RAr-Pe; RF-Pe; RP-Pe; RV-Pe; SE-Pe; VC-Pe; RF-RAr; RV-RAr; SE-RAr; RP-RF; RV-RP; SE-RP; VC-RP; VC-RV; VC-SE
DGDG (34:6)	1.75E-16	RAv-Al; Pe-Al; RAr-Al; RP-Al; VC-Al; Pe-RAv; RAr-RAv; RF-RAv; RP-RAv; RV-RAv; VC-RAv; RAr-Pe; RF-Pe; RV-Pe; SE-Pe; RF-RAr; SE-RAr; RP-RF; VC-RF; RV-RP; SE-RP; SE-RV; VC-RV; VC-SE
PC (34:4)	5.27E-16	RP-Al; VC-Al; RP-RAv; RP-Pe; RP-RAr; RP-RF; VC-RF; RV-RP; SE-RP; VC-RP; VC-RV
DGTS (42:10)	1.16E-15	RAv-Al; SE-Al; Pe-RAv; RAr-RAv; RF-RAv; RP-RAv; RV-RAv; SE-RAv; VC-RAv; RV-Pe; SE-Pe; RV-RAr; SE-RAr; SE-RF; RV-RP; SE-RP; SE-RV; VC-RV; VC-SE
DGTS (36:3)	1.59E-15	Pe-Al; RAr-Al; RP-Al; VC-Al; Pe-RAv; RAr-RAv; RP-RAv; RV-RAv; VC-RAv; RF-Pe; RV-Pe; SE-Pe; RF-RAr; RV-RAr; SE-RAr; RP-RF; VC-RF; RV-RP; SE-RP; SE-RV; VC-RV; VC-SE
DGTS (38:8)	1.63E-15	RAv-Al; Pe-Al; RP-Al; SE-Al; Pe-RAv; RAr-RAv; RF-RAv; RP-RAv; RV-RAv; SE-RAv; VC-RAv; RF-Pe; RV-Pe; SE-Pe; RF-RAr; RV-RAr; SE-RAr; RP-RF; SE-RF; VC-RF; RV-RP; SE-RP; SE-RV; VC-RV; VC-SE
MGDG (34:6)	1.74E-15	Pe-Al; RP-Al; SE-Al; VC-Al; Pe-RAv; RAr-RAv; RF-RAv; RP-RAv; RV-RAv; VC-RAv; RAr-Pe; RF-Pe; RP-Pe; RV-Pe; SE-Pe; VC-Pe; RP-RAr; SE-RAr; VC-RAr; RP-RF; SE-RF; VC-RF; RV-RP; SE-RP; SE-RV; VC-RV; VC-SE
PC (34:3)	5.10E-15	RAv-Al; RAr-Al; RP-Al; SE-Al; VC-Al; Pe-RAv; RF-RAv; RP-RAv; RV-RAv; RP-Pe; VC-Pe; RP-RAr; RP-RF; VC-RF; RV-RP; SE-RP; VC-RP; VC-RV
MGDG (36:8)	6.56E-15	RAv-Al; RAr-Al; RP-Al; SE-Al; VC-Al; Pe-RAv; RAr-RAv; RF-RAv; RP-RAv; RV-RAv; SE-RAv; VC-RAv; RF-Pe; RF-RAr; RP-RF; RV-RF; SE-RF; VC-RF; RV-RP; SE-RV
DGTS (40:9)	1.26E-14	RAv-Al; SE-Al; Pe-RAv; RAr-RAv; RF-RAv; RP-RAv; RV-RAv; VC-RAv; RF-Pe; RV-Pe; SE-Pe; RV-RAr; SE-RAr; SE-RF; RV-RP; SE-RP; SE-RV; VC-SE
MGTS (16:4)	1.34E-14	RAv-Al; SE-Al; VC-Al; SE-RAv; SE-Pe; SE-RAr; VC-RAr; SE-RF; VC-RF; SE-RP; VC-RP; SE-RV; VC-RV; VC-SE
DGTS (36:5)	1.54E-14	RAv-Al; SE-Al; Pe-RAv; RAr-RAv; RF-RAv; RP-RAv; RV-RAv; VC-RAv; RV-Pe; SE-Pe; RV-RAr; SE-RAr; SE-RF; SE-RP; SE-RV; VC-SE
DGTS (38:7)	2.39E-14	RAv-Al; Pe-Al; RAr-Al; RP-Al; SE-Al; VC-Al; Pe-RAv; RAr-RAv; RF-RAv; RP-RAv; RV-RAv; SE-RAv; VC-RAv; RF-Pe; RV-Pe; SE-Pe; RF-RAr; RV-RAr; SE-RAr; RP-RF; VC-RF; RV-RP; SE-RP; VC-RV; VC-SE
MGTS (14:0)	2.71E-14	Pe-Al; RAr-Al; RP-Al; RV-Al; VC-Al; Pe-RAv; RAr-RAv; RP-RAv; RV-RAv; SE-RAv; VC-RAv; RF-Pe; SE-Pe; RV-RAr; VC-RAr; RP-RF; RV-RF; VC-RF; RV-RP; SE-RP; VC-RP; SE-RV; VC-SE
MGMG (16:3)	3.88E-14	RF-Al; RV-Al; RF-RAv; RV-RAv; SE-RAv; RAr-Pe; RV-Pe; SE-Pe; RF-RAr; RV-RAr; VC-

		RAr; RP-RF; RV-RF; SE-RF; VC-RF; RV-RP; SE-RP; SE-RV; VC-RV; VC-SE
DGDG (36:6)	4.29E-14	Pe-Al; RAr-Al; RF-Al; RP-Al; VC-Al; Pe-RAv; RAr-RAv; RF-RAv; RP-RAv; SE-RAv; VC-RAv; RV-Pe; SE-Pe; RV-RAr; SE-RAr; RV-RF; SE-RF; RV-RP; SE-RP; VC-RV; VC-SE
PC (40:8)	4.29E-14	RAv-Al; RAr-Al; RF-Al; RP-Al; SE-Al; VC-Al; Pe-RAv; RAr-RAv; RF-RAv; RV-RAv; RP-Pe; SE-Pe; RP-RAr; RV-RAr; SE-RAr; RP-RF; RV-RF; SE-RF; RV-RP; VC-RP; SE-RV; VC-RV; VC-SE
DGTS (38:4)	4.70E-14	RAv-Al; RP-Al; SE-Al; Pe-RAv; RAr-RAv; RF-RAv; RP-RAv; RV-RAv; VC-RAv; RF-Pe; SE-Pe; RF-RAr; SE-RAr; RP-RF; SE-RF; VC-RF; RV-RP; SE-RP; SE-RV; VC-SE
PC (38:7)	6.25E-14	RAv-Al; RP-Al; SE-Al; VC-Al; Pe-RAv; RAr-RAv; RF-RAv; RV-RAv; VC-RAv; RP-Pe; SE-Pe; VC-Pe; RP-RAr; SE-RAr; RP-RF; SE-RF; VC-RF; RV-RP; SE-RP; VC-RP; SE-RV; VC-RV
DGTS (30:0)	7.42E-14	Pe-Al; RP-Al; Pe-RAv; RAr-RAv; RP-RAv; VC-RAv; RAr-Pe; RF-Pe; RP-Pe; RV-Pe; SE-Pe; VC-Pe; RP-RAr; RP-RF; RV-RP; SE-RP; VC-RP; VC-SE
MGTS (16:1)	7.42E-14	RAv-Al; Pe-Al; RAr-Al; RP-Al; RV-Al; SE-Al; VC-Al; Pe-RAv; RF-RAv; RV-RAv; VC-RAv; RF-Pe; RF-RAr; VC-RAr; RP-RF; RV-RF; SE-RF; VC-RF; VC-RP; VC-RV; VC-SE
DGTS (34:8)	7.42E-14	RAv-Al; RF-Al; SE-Al; VC-Al; Pe-RAv; RAr-RAv; RP-RAv; RV-RAv; RF-Pe; RP-Pe; RV-Pe; SE-Pe; VC-Pe; RF-RAr; SE-RAr; VC-RAr; SE-RF; VC-RF; SE-RP; VC-RP; SE-RV; VC-RV
DGTS (34:6)	8.43E-14	RAv-Al; Pe-Al; SE-Al; Pe-RAv; RAr-RAv; RF-RAv; RP-RAv; RV-RAv; VC-RAv; RAr-Pe; RF-Pe; RP-Pe; RV-Pe; SE-Pe; VC-Pe; SE-RAr; SE-RF; SE-RP; SE-RV; VC-SE
PE (32:0)	1.02E-13	Pe-Al; RAr-Al; RP-Al; SE-Al; VC-Al; Pe-RAv; RAr-RAv; RP-RAv; SE-RAv; VC-RAv; RF-Pe; RV-Pe; RF-RAr; RV-RAr; RP-RF; SE-RF; VC-RF; RV-RP; VC-RP; SE-RV; VC-RV
MGDG (32:1)	1.02E-13	RAv-Al; Pe-Al; RAr-Al; RF-Al; RP-Al; RV-Al; SE-Al; VC-Al; RAr-RAv; RF-RAv; RV-RAv; RF-Pe; RF-RAr; RP-RAr; RP-RF; RV-RF; SE-RF; VC-RF; RV-RP
PE (34:3)	2.30E-13	RAv-Al; RF-Al; RP-Al; RV-Al; SE-Al; RP-RAv; RV-RAv; SE-RAv; RP-Pe; RV-Pe; SE-Pe; RP-RAr; RV-RAr; SE-RAr; RV-RF; SE-RF; RV-RP; VC-RP; SE-RV; VC-RV; VC-SE
MGDG (34:2)	2.36E-13	RAv-Al; RP-Al; SE-Al; Pe-RAv; RAr-RAv; RF-RAv; RP-RAv; RV-RAv; SE-RAv; VC-RAv; RV-Pe; SE-Pe; RP-RAr; SE-RAr; RP-RF; SE-RF; RV-RP; SE-RP; VC-RP; SE-RV; VC-SE
DGTS (38:6)	2.81E-13	RAv-Al; RP-Al; SE-Al; VC-Al; Pe-RAv; RAr-RAv; RF-RAv; RP-RAv; RV-RAv; VC-RAv; RV-Pe; SE-Pe; RV-RAr; SE-RAr; RP-RF; SE-RF; VC-RF; RV-RP; SE-RP; SE-RV; VC-RV; VC-SE
DGDG (32:3)	4.32E-13	RAv-Al; SE-Al; Pe-RAv; RAr-RAv; RF-RAv; RP-RAv; RV-RAv; VC-RAv; RV-Pe; SE-Pe; RV-RAr; SE-RAr; RV-RF; SE-RF; SE-RP; SE-RV; VC-SE
DGTS (38:10)	4.37E-13	RAv-Al; Pe-Al; RP-Al; SE-Al; Pe-RAv; RAr-RAv; RF-RAv; RP-RAv; RV-RAv; SE-RAv; VC-RAv; RAr-Pe; RF-Pe; SE-Pe; VC-Pe; RP-RAr; RP-RF; RV-RF; SE-RP; SE-RV; VC-SE
MGTS (16:2)	6.01E-13	RAv-Al; Pe-Al; RP-Al; SE-Al; VC-Al; RAr-RAv; RF-RAv; SE-RAv; RAr-Pe; RF-Pe; RV-Pe; RF-RAr; SE-RAr; VC-RAr; RP-RF; RV-RF; SE-RF; VC-RF; SE-RP; VC-RP; SE-RV; VC-RV
MGDG (34:1)	6.01E-13	RAv-Al; Pe-Al; RAr-Al; RP-Al; RV-Al; SE-Al; VC-Al; Pe-RAv; RAr-RAv; RF-RAv; RP-RAv; RV-RAv; SE-RAv; VC-RAv; RF-Pe; SE-Pe; RF-RAr; RP-RF; RV-RF; SE-RF; VC-RF; SE-RP; SE-RV
DGDG (36:7)	6.26E-13	RAv-Al; Pe-Al; RF-Al; RV-Al; Pe-RAv; RAr-RAv; RF-RAv; RP-RAv; SE-RAv; VC-RAv; RAr-Pe; RP-Pe; RV-Pe; SE-Pe; RF-RAr; RV-RAr; RP-RF; RV-RF; SE-RF; VC-RF; RV-RP; VC-RV; VC-SE
MGDG (38:9)	8.36E-13	RAv-Al; Pe-Al; RAr-Al; RF-Al; RP-Al; SE-Al; VC-Al; Pe-RAv; RAr-RAv; RF-RAv; RP-RAv; RV-RAv; VC-RAv; RF-Pe; RV-Pe; SE-Pe; RV-RAr; SE-RAr; RP-RF; SE-RF; RV-RP; SE-RP; SE-RV; VC-SE
MGMG (16:1)	8.36E-13	Pe-Al; RP-Al; RV-Al; Pe-RAv; RP-RAv; RV-RAv; RAr-Pe; RF-Pe; RV-Pe; SE-Pe; VC-Pe; RF-RAr; RP-RAr; RV-RAr; RP-RF; SE-RF; VC-RF; RV-RP; SE-RP; VC-RP; SE-RV; VC-RV
PC (38:5)	1.69E-12	RP-Al; RP-RAv; RP-Pe; RP-RAr; RP-RF; RV-RP; SE-RP; VC-RP
MGMG (16:2)	1.80E-12	RAr-Al; RP-Al; RV-Al; SE-Al; RAr-RAv; RP-RAv; RV-RAv; SE-RAv; RF-Pe; RP-Pe; RV-Pe; SE-Pe; RF-RAr; RV-RAr; RP-RF; SE-RF; VC-RF; RV-RP; VC-RP; SE-RV; VC-RV; VC-SE
DGTS (38:9)	1.92E-12	RAv-Al; Pe-RAv; RAr-RAv; RF-RAv; RP-RAv; RV-RAv; SE-RAv; VC-RAv; RF-Pe; RV-Pe; SE-Pe; SE-RAr; RP-RF; RV-RP; SE-RP
PG (36:5)	3.30E-12	RAv-Al; Pe-Al; RP-Al; SE-Al; VC-Al; RF-RAv; RP-RAv; SE-RAv; RF-Pe; RP-Pe; RF-RAr; RP-RAr; SE-RAr; VC-RAr; RP-RF; RV-RF; SE-RF; VC-RF; RV-RP; VC-RP; SE-RV
MGTS (18:4)	3.44E-12	RAv-Al; Pe-Al; RAr-Al; RP-Al; SE-Al; VC-Al; RAr-RAv; RF-RAv; RV-RAv; RAr-Pe; RF-Pe; RV-Pe; SE-RAr; VC-RAr; RP-RF; SE-RF; VC-RF; RV-RP; SE-RV; VC-RV
PE (40:8)	4.21E-12	RAv-Al; RP-Al; SE-Al; Pe-RAv; RAr-RAv; RF-RAv; RV-RAv; VC-RAv; RP-Pe; SE-Pe; RP-RAr; SE-RAr; RP-RF; SE-RF; RV-RP; VC-RP; SE-RV; VC-SE

MGTS (22:5)	4.66E-12	RAv-Al; Pe-Al; RAr-Al; RP-Al; SE-Al; VC-Al; RAr-RAv; RF-RAv; RP-RAv; RV-RAv; RAr-Pe; RF-Pe; RP-Pe; RV-Pe; SE-RAr; VC-RAr; SE-RF; VC-RF; RV-RP; SE-RP; SE-RV; VC-RV
MGTS (16:3)	4.97E-12	RAv-Al; Pe-Al; RAr-Al; RP-Al; SE-Al; VC-Al; RF-RAv; RV-RAv; VC-RAv; RF-Pe; RV-Pe; VC-Pe; RF-RAr; RV-RAr; RP-RF; SE-RF; VC-RF; RV-RP; VC-RP; SE-RV; VC-RV
PG (32:0)	5.71E-12	Pe-Al; RAr-Al; RP-Al; RV-Al; SE-Al; VC-Al; Pe-RAv; RAr-RAv; RP-RAv; RV-RAv; SE-RAv; VC-RAv; RF-Pe; RP-Pe; RF-RAr; RP-RAr; RP-RF; RV-RF; SE-RF; VC-RF; RV-RP; SE-RP; VC-RP
MGDG (34:4)	5.71E-12	Pe-Al; Pe-RAv; RF-RAv; VC-RAv; RAr-Pe; RF-Pe; RP-Pe; RV-Pe; SE-Pe; VC-Pe; RV-RF; SE-RF; VC-RV; VC-SE
DGTS (34:2)	5.80E-12	RAv-Al; Pe-Al; RAr-Al; RP-Al; SE-Al; VC-Al; Pe-RAv; RAr-RAv; RF-RAv; RP-RAv; RV-RAv; VC-RAv; RF-Pe; RV-Pe; SE-Pe; RV-RAr; SE-RAr; RP-RF; SE-RF; RV-RP; SE-RP; SE-RV; VC-RV; VC-SE
MGDG (32:6)	8.07E-12	Pe-Al; RP-Al; SE-Al; VC-Al; Pe-RAv; RP-RAv; SE-RAv; RAr-Pe; RF-Pe; RP-Pe; RV-Pe; SE-Pe; VC-Pe; RP-RAr; SE-RAr; RP-RF; SE-RF; VC-RF; RV-RP
MGDG (32:2)	1.18E-11	RF-Al; RP-Al; SE-Al; RF-RAv; SE-RAv; VC-RAv; RF-Pe; RP-Pe; SE-Pe; RF-RAr; RP-RAr; SE-RAr; RP-RF; RV-RF; SE-RF; VC-RF; RV-RP; VC-RP; SE-RV; VC-SE
PE (36:5)	1.18E-11	RP-Al; SE-Al; RP-RArrv; RV-RArrv; SE-RArrv; RP-Pe; RV-Pe; SE-Pe; RP-RArr; RV-RArr; SE-RArr; RP-RF; RV-RF; SE-RF; RV-RP; VC-RP; SE-RV; VC-RV; VC-SE
PC (32:2)	1.56E-11	Pe-Al; RP-Al; Pe-RAv; RP-RAv; RF-Pe; RP-Pe; RV-Pe; SE-Pe; RP-RAr; RP-RF; RV-RP; SE-RP; VC-RP
SQDG (32:3)	1.82E-11	RAv-Al; RV-Al; SE-Al; Pe-RAv; RAr-RAv; RF-RAv; RP-RAv; VC-RAv; RF-Pe; RP-Pe; RV-Pe; SE-Pe; RV-RAr; SE-RAr; RV-RF; SE-RF; SE-RP; VC-RV; VC-SE
DGTS (34:7)	2.05E-11	RAv-Al; SE-Al; Pe-RAv; RAr-RAv; RF-RAv; RP-RAv; RV-RAv; VC-RAv; RV-Pe; SE-Pe; VC-Pe; SE-RAr; SE-RF; SE-RP; SE-RV; VC-SE
DGTS (36:7)	2.17E-11	RAv-Al; SE-Al; Pe-RAv; RAr-RAv; RF-RAv; RP-RAv; RV-RAv; VC-RAv; SE-Pe; SE-RAr; SE-RF; SE-RP; SE-RV; VC-SE
DGTS (40:4)	2.27E-11	RAv-Al; RV-Al; SE-Al; Pe-RAv; RAr-RAv; RF-RAv; RP-RAv; RV-RAv; VC-RAv; SE-Pe; SE-RAr; SE-RF; SE-RP; SE-RV; VC-SE
PC (34:2)	2.27E-11	RP-Al; RP-RAv; RP-Pe; VC-Pe; RP-RAr; RP-RF; RV-RP; SE-RP; VC-RP
LPE (20:4)	2.49E-11	RAv-Al; RV-Al; SE-Al; RF-RAv; RV-RAv; SE-RAv; RV-Pe; SE-Pe; RV-RAr; SE-RAr; RV-RF; SE-RF; RV-RP; SE-RP; SE-RV; VC-RV; VC-SE
DGDG (32:4)	3.87E-11	RAv-Al; RV-Al; VC-Al; Pe-RAv; RAr-RAv; RF-RAv; RP-RAv; RV-RAv; SE-RAv; RV-Pe; VC-Pe; RV-RAr; VC-RAr; RP-RF; RV-RF; VC-RF; VC-SE
PC (36:6)	3.89E-11	RAv-Al; RP-Al; VC-Al; Pe-RAv; RAr-RAv; RF-RAv; RP-RAv; RV-RAv; SE-RAv; RP-Pe; VC-Pe; RP-RAr; VC-RAr; RP-RF; VC-RF; RV-RP; SE-RP; VC-RP; VC-RV; VC-SE
MGTS (18:3)	3.89E-11	RAv-Al; Pe-Al; RAr-Al; RP-Al; SE-Al; VC-Al; RF-RAv; RV-RAv; RAr-Pe; RF-Pe; RV-Pe; RF-RAr; VC-RAr; RP-RF; SE-RF; VC-RF; VC-RP; SE-RV; VC-RV; VC-SE
MGMG (18:3)	3.95E-11	Pe-Al; RP-Al; Pe-RAv; RAr-RAv; RP-RAv; RF-Pe; RV-Pe; SE-Pe; VC-Pe; RP-RAr; SE-RAr; VC-RAr; RP-RF; RV-RP; SE-RP; VC-RP
PE3 (2:2)	3.98E-11	RP-Al; SE-Al; RP-RAv; RV-RAv; SE-RAv; RP-Pe; RV-Pe; RP-RAr; RV-RAr; RP-RF; RV-RF; RV-RP; SE-RP; VC-RP; SE-RV; VC-RV
MGDG (34:3)	4.43E-11	RAv-Al; RAr-Al; RV-Al; SE-Al; VC-Al; Pe-RAv; RAr-RAv; RF-RAv; RP-RAv; VC-RAv; RAr-Pe; RV-Pe; SE-Pe; VC-Pe; RP-RAr; RV-RF; SE-RF; RV-RP; SE-RP; VC-RP
PE (32:1)	4.59E-11	Pe-Al; RP-Al; SE-Al; Pe-RAv; RP-RAv; SE-RAv; RF-Pe; RP-Pe; RV-Pe; VC-Pe; RP-RAr; RP-RF; SE-RF; RV-RP; SE-RP; VC-RP; SE-RV
DGTS (36:4)	4.65E-11	RAv-Al; Pe-Al; SE-Al; Pe-RAv; RAr-RAv; RF-RAv; RP-RAv; RV-RAv; VC-RAv; RF-Pe; RV-Pe; SE-Pe; RV-RAr; SE-RAr; SE-RF; RV-RP; SE-RP; VC-RV; VC-SE
MGDG (34:8)	5.50E-11	RAv-Al; Pe-Al; RV-Al; Pe-RAv; RAr-RAv; RF-RAv; RP-RAv; SE-RAv; VC-RAv; RAr-Pe; RF-Pe; RV-Pe; RP-RAr; VC-RAr; RV-RF; RV-RP; SE-RV; VC-RV
MGMG (18:1)	6.91E-11	Pe-Al; RP-Al; RV-Al; VC-Al; Pe-RAv; RV-RAv; VC-RAv; RF-Pe; SE-Pe; RV-RAr; VC-RAr; RP-RF; RV-RF; VC-RF; SE-RP; VC-RP; SE-RV; VC-SE
MGDG (36:7)	8.68E-11	RAv-Al; SE-Al; Pe-RAv; RAr-RAv; RF-RAv; RP-RAv; RV-RAv; VC-RAv; SE-Pe; SE-RAr; RP-RF; SE-RF; SE-RV; VC-SE
PC (38:6)	1.09E-10	RAv-Al; RP-Al; SE-Al; RAr-RAv; RF-RAv; RP-RAv; RP-Pe; RP-RAr; RP-RF; RV-RP; SE-RP; VC-RP
PE (30:1)	1.10E-10	RAv-Al; RV-Al; VC-Al; Pe-RAv; RAr-RAv; RF-RAv; RP-RAv; SE-RAv; RP-Pe; RV-Pe; RP-RAr; RV-RAr; RV-RF; VC-RF; RV-RP; SE-RP; VC-RP; SE-RV; VC-RV
DGMG (16:0)	1.20E-10	Pe-Al; RAr-Al; RP-Al; VC-Al; Pe-RAv; RF-RAv; VC-RAv; RAr-Pe; RF-Pe; RP-Pe; RV-Pe; SE-

		Pe; RF-RAr; RP-RF; SE-RF; VC-RF; VC-RV; VC-SE
DGDG (32:2)	1.23E-10	RF-Al; Pe-RAv; RAr-RAv; RF-RAv; RV-RAv; VC-RAv; RF-Pe; RF-RAr; SE-RAr; RP-RF; RV-RF; SE-RF; VC-RF; VC-SE
PE (36:2)	1.26E-10	Pe-Al; RP-Al; SE-Al; Pe-RAv; RP-RAv; SE-RAv; RAr-Pe; RV-Pe; VC-Pe; RP-RAr; SE-RAr; RP-RF; RV-RF; RV-RP; SE-RP; VC-RP; SE-RV; VC-SE
DGTS (30:2)	1.38E-10	Pe-Al; RF-Al; RV-Al; Pe-RAv; RF-RAv; RV-RAv; RAr-Pe; RP-Pe; RV-Pe; SE-Pe; VC-Pe; RF-RAr; RV-RAr; RP-RF; RV-RF; SE-RF; VC-RF; RV-RP; SE-RV; VC-RV
PE (36:4)	1.40E-10	RAr-Al; RP-Al; SE-Al; RP-RAv; RV-RAv; RP-Pe; RV-Pe; SE-Pe; RP-RAr; RV-RAr; RP-RF; RV-RF; SE-RF; RV-RP; VC-RP; SE-RV; VC-RV; VC-SE
DGTS (34:3)	1.48E-10	RAv-Al; SE-Al; Pe-RAv; RAr-RAv; RF-RAv; RP-RAv; RV-RAv; VC-RAv; SE-Pe; SE-RAr; SE-RF; RV-RP; SE-RP; SE-RV; VC-SE
MGTS (20:5)	1.48E-10	RAv-Al; Pe-Al; RAr-Al; SE-Al; VC-Al; RF-RAv; RV-RAv; RF-Pe; RP-Pe; RV-Pe; RF-RAr; RV-RAr; VC-RAr; SE-RF; VC-RF; SE-RP; VC-RP; SE-RV; VC-RV
PC (38:4)	2.06E-10	RP-Al; RP-RAv; RP-Pe; VC-Pe; RP-RAr; RP-RF; VC-RF; RV-RP; SE-RP; VC-RP
LPC (22:6)	2.60E-10	RAv-Al; RF-Al; RP-Al; Pe-RAv; RP-RAv; RV-RAv; VC-RAv; RF-Pe; RP-Pe; RP-RAr; RV-RAr; VC-RAr; RP-RF; RV-RF; VC-RF; SE-RP; SE-RV; VC-SE
PI (34:2)	4.07E-10	Pe-Al; RAr-Al; VC-Al; Pe-RAv; RAr-RAv; VC-RAv; RF-Pe; RP-Pe; SE-Pe; RF-RAr; RP-RAr; RV-RAr; SE-RAr; SE-RF; VC-RF; VC-RP; SE-RV; VC-RV; VC-SE
MGTS (18:2)	4.07E-10	RAv-Al; Pe-Al; RAr-Al; SE-Al; VC-Al; RF-RAv; RP-RAv; RV-RAv; RF-Pe; RV-Pe; RF-RAr; RP-RAr; RV-RAr; SE-RF; VC-RF; SE-RP; VC-RP; SE-RV; VC-RV
PE (34:1)	4.08E-10	Pe-Al; RP-Al; SE-Al; Pe-RAv; RP-RAv; SE-RAv; RF-Pe; RV-Pe; VC-Pe; RP-RAr; RV-RAr; SE-RAr; RP-RF; SE-RF; RV-RP; VC-RP; SE-RV; VC-SE
LPC (18:1)	4.38E-10	RP-Al; RV-Al; RP-RAv; RV-RAv; VC-RAv; RP-Pe; SE-Pe; RP-RAr; RV-RAr; RP-RF; RV-RF; SE-RP; VC-RP; SE-RV; VC-SE
PC (34:1)	4.90E-10	RP-Al; RP-RAv; RP-Pe; RP-RAr; RP-RF; RV-RP; SE-RP; VC-RP
PC (32:0)	4.96E-10	RP-Al; VC-Al; RP-RAv; RV-RAv; VC-RAv; RP-Pe; RF-RAr; RP-RAr; SE-RAr; RP-RF; RV-RF; VC-RF; RV-RP; SE-RP; VC-RP; SE-RV; VC-SE
DGDG (32:0)	5.84E-10	RAv-Al; Pe-Al; RAr-Al; RP-Al; RV-Al; VC-Al; RF-RAv; RF-Pe; RF-RAr; RP-RF; RV-RF; SE-RF; VC-RF; VC-RV; VC-SE
MGTS (16:0)	7.83E-10	RV-Al; VC-Al; RV-RAv; VC-RAv; RV-Pe; VC-Pe; RV-RAr; VC-RAr; RV-RF; VC-RF; RV-RP; VC-RP; SE-RV; VC-SE
MGDG (36:4)	8.19E-10	Pe-Al; Pe-RAv; RAr-RAv; RP-RAv; VC-RAv; RAr-Pe; RF-Pe; RP-Pe; RV-Pe; SE-Pe; RF-RAr; RV-RAr; RP-RF; VC-RF; RV-RP; VC-RV; VC-SE
MGMG (16:0)	8.37E-10	RAv-Al; Pe-Al; RP-Al; RV-Al; SE-Al; VC-Al; RF-RAv; RF-Pe; RF-RAr; VC-RAr; RP-RF; RV-RF; SE-RF; VC-RF; VC-RP
PC (36:5)	9.49E-10	RAv-Al; Pe-Al; RAr-Al; RP-Al; RV-Al; SE-Al; VC-Al; RF-RAv; RP-RAv; RP-Pe; RP-RAr; RP-RF; RV-RP; SE-RP; VC-RP
MGTS (22:6)	9.79E-10	RAv-Al; SE-Al; VC-Al; Pe-RAv; RAr-RAv; RF-RAv; RP-RAv; RV-RAv; SE-Pe; VC-Pe; SE-RF; VC-RF; SE-RP; VC-RP; SE-RV
PC (36:3)	9.94E-10	RAv-Al; RP-Al; VC-Al; Pe-RAv; RAr-RAv; RV-RAv; VC-RAv; RP-Pe; VC-Pe; RF-RAr; RP-RAr; SE-RAr; RV-RF; VC-RF; RV-RP; SE-RP; VC-RP; VC-SE
PG (34:2)	1.61E-09	Pe-Al; RAr-Al; RP-Al; RV-Al; SE-Al; VC-Al; RP-RAv; SE-RAv; RF-Pe; RP-Pe; SE-Pe; RF-RAr; RP-RAr; SE-RAr; RP-RF; RV-RF; SE-RF; VC-RF; RV-RP; VC-RP; SE-RV; VC-SE
DGTS (36:8)	1.90E-09	RAv-Al; SE-Al; Pe-RAv; RAr-RAv; RF-RAv; RP-RAv; RV-RAv; VC-RAv; SE-Pe; SE-RAr; SE-RF; SE-RP; SE-RV; VC-SE
PE (36:3)	2.00E-09	RAv-Al; RF-Al; RP-Al; SE-Al; RAr-RAv; RV-RAv; VC-RAv; RV-Pe; VC-Pe; RF-RAr; RP-RAr; SE-RAr; RV-RF; VC-RF; RV-RP; VC-RP; SE-RV; VC-SE
MGDG (32:4)	2.90E-09	RV-Al; VC-Al; Pe-RAv; RF-RAv; RAr-Pe; RV-Pe; SE-Pe; VC-Pe; RF-RAr; RV-RF; SE-RF; VC-RF; RV-RP; VC-RP
MGDG (32:5)	3.24E-09	RV-Al; Pe-RAv; RF-RAv; RV-RAv; RAr-Pe; RP-Pe; RV-Pe; SE-Pe; VC-Pe; RF-RAr; RP-RF; RV-RF; SE-RF; VC-RF; SE-RV
MGMG (16:4)	3.87E-09	RAv-Al; Pe-Al; RF-Al; RP-Al; RV-Al; VC-Al; RAr-RAv; RV-RAv; SE-RAv; RAr-Pe; RV-Pe; SE-Pe; RF-RAr; RP-RAr; RV-RAr; VC-RAr; RV-RF; SE-RF; RV-RP; SE-RP; SE-RV; VC-SE
PC (36:2)	4.55E-09	RP-Al; RP-RAv; RAr-Pe; RP-Pe; VC-Pe; RF-RAr; RP-RAr; RP-RF; VC-RF; RV-RP; SE-RP; VC-RP
PE (34:2)	5.58E-09	RP-Al; RV-Al; RP-RAv; RV-RAv; RP-Pe; RV-Pe; RP-RAr; RV-RAr; RP-RF; RV-RF; RV-RP; VC-RP; SE-RV; VC-RV; VC-SE

SQDG (32:4)	5.92E-09	RV-Al; SE-Al; RV-RAv; SE-RAv; RP-Pe; RV-Pe; SE-Pe; VC-Pe; RV-RAr; SE-RAr; RP-RF; RV-RF; SE-RF; VC-RF; RV-RP; SE-RP
DGDG (32:1)	7.13E-09	RF-Al; RF-RAv; RF-Pe; RF-RAr; RP-RF; RV-RF; SE-RF; VC-RF; SE-RP
DGTS (38:5)	9.60E-09	RAv-Al; Pe-Al; SE-Al; Pe-RAv; RAr-RAv; RF-RAv; RP-RAv; RV-RAv; VC-RAv; SE-Pe; VC-Pe; SE-RAr; SE-RF; SE-RP; SE-RV; VC-SE
DGDG (36:2)	1.00E-08	Pe-Al; RAr-Al; SE-Al; VC-Al; Pe-RAv; RAr-RAv; RF-Pe; RP-Pe; RV-Pe; RP-RAr; RV-RAr; RV-RF; VC-RP; SE-RV; VC-RV
DGDG (34:5)	1.03E-08	Pe-Al; RAr-Al; RP-Al; VC-Al; Pe-RAv; RAr-RAv; RP-RAv; VC-RAv; RF-Pe; RP-Pe; RV-Pe; SE-Pe; SE-RAr; VC-RF; VC-SE
MGTS (18:1)	1.45E-08	RAv-Al; Pe-Al; RAr-Al; RP-Al; RV-Al; SE-Al; VC-Al; RF-RAv; RF-Pe; VC-Pe; RF-RAr; RP-RF; RV-RF; SE-RF; VC-RF; VC-SE
SQDG (30:0)	1.76E-08	RP-Al; RV-Al; SE-Al; RF-RAv; SE-RAv; RF-Pe; SE-Pe; RF-RAr; SE-RAr; RP-RF; RV-RF; SE-RF; VC-RF
SQDG (36:1)	2.22E-08	RV-Al; SE-Al; Pe-RAv; RAr-RAv; RP-RAv; VC-RAv; RV-Pe; SE-Pe; RV-RAr; SE-RAr; SE-RF; RV-RP; SE-RP; VC-RV; VC-SE
PG (34:4)	2.69E-08	RV-Al; SE-Al; Pe-RAv; RV-RAv; SE-RAv; RF-Pe; RP-Pe; RV-Pe; SE-Pe; RV-RAr; SE-RAr; RV-RF; SE-RF; RV-RP; SE-RP; VC-RV; VC-SE
PI (42:11)	2.98E-08	Pe-Al; RP-Al; Pe-RAv; RAr-RAv; RP-RAv; VC-RAv; RF-Pe; RV-Pe; SE-Pe; RV-RAr; RV-RF; RV-RP; SE-RP; VC-RV
DGTS (36:6)	3.88E-08	RAv-Al; SE-Al; Pe-RAv; RAr-RAv; RF-RAv; RP-RAv; RV-RAv; VC-RAv; SE-Pe; SE-RAr; SE-RF; SE-RP; SE-RV; VC-SE
MGDG (34:5)	4.97E-08	Pe-Al; Pe-RAv; RF-RAv; VC-RAv; RAr-Pe; RF-Pe; RP-Pe; RV-Pe; SE-Pe; VC-Pe; RF-RAr; VC-RAr
SQMG (16:0)	6.21E-08	SE-Al; VC-Al; Pe-RAv; RAr-RAv; RP-RAv; SE-RAv; VC-RAv; SE-RF; VC-RF; VC-RP; SE-RV; VC-RV
DGTS (34:5)	6.55E-08	RAv-Al; SE-Al; Pe-RAv; RAr-RAv; RF-RAv; RP-RAv; VC-RAv; RV-Pe; SE-Pe; SE-RAr; RV-RF; SE-RF; SE-RP; VC-SE
MGTS (20:4)	7.06E-08	RAv-Al; SE-Al; VC-Al; RF-RAv; VC-Pe; VC-RAr; SE-RF; VC-RF; VC-RP; VC-RV
PG (32:1)	7.93E-08	RP-Al; SE-Al; Pe-RAv; RAr-RAv; RP-RAv; RV-RAv; SE-RAv; RF-Pe; RP-Pe; RF-RAr; RP-RF; RV-RF; SE-RF; VC-RF; VC-RP
DGDG (28:0)	8.45E-08	RAv-Al; RF-Al; RP-Al; RF-RAv; RF-Pe; RF-RAr; RP-RF; RV-RF; SE-RF; VC-RF; SE-RP
DGTS (34:4)	8.52E-08	RAv-Al; SE-Al; Pe-RAv; RAr-RAv; RF-RAv; RP-RAv; RV-RAv; VC-RAv; SE-Pe; SE-RAr; SE-RF; SE-RP; SE-RV; VC-SE
SQDG (36:3)	8.53E-08	RAv-Al; SE-Al; Pe-RAv; RAr-RAv; RF-RAv; RP-RAv; VC-RAv; RV-Pe; SE-Pe; SE-RAr; SE-RF; RV-RP; SE-RP; VC-RV; VC-SE
DGDG (36:4)	1.81E-07	Pe-Al; RAr-Al; RF-Al; RP-Al; SE-Al; VC-Al; Pe-RAv; RAr-RAv; RF-RAv; VC-RAv; RV-Pe; RV-RAr; RV-RF; VC-RV
DGTS (32:0)	1.90E-07	RP-Al; RV-Al; VC-Al; RV-RAv; SE-RAv; SE-Pe; RV-RAr; SE-RAr; RP-RF; RV-RF; SE-RP; SE-RV; VC-SE
PG (34:3)	2.03E-07	RV-Al; SE-Al; RV-RAv; SE-RAv; RAr-Pe; RF-Pe; RP-Pe; RV-Pe; SE-Pe; SE-RAr; VC-RF; SE-RP; VC-RV; VC-SE
DGTS (32:5)	2.55E-07	RAv-Al; SE-Al; Pe-RAv; RAr-RAv; RF-RAv; RP-RAv; RV-Pe; SE-Pe; VC-Pe; SE-RAr; SE-RF; SE-RP
PC (36:4)	3.08E-07	RAv-Al; Pe-Al; RAr-Al; RP-Al; VC-Al; RP-RAv; RP-Pe; RP-RAr; RP-RF; RV-RP; SE-RP; VC-RP
MGDG (36:5)	6.71E-07	RP-Al; SE-Al; Pe-RAv; RF-RAv; RP-Pe; SE-Pe; SE-RAr; RP-RF; SE-RF; SE-RV; VC-SE
SQDG (32:0)	6.92E-07	RV-Al; SE-Al; RV-RAv; SE-RAv; RV-Pe; SE-Pe; RV-RAr; SE-RAr; RV-RF; SE-RF; SE-RP; VC-SE
PC (40:9)	7.54E-07	RAv-Al; RAr-Al; RF-Al; RP-Al; SE-Al; VC-Al; RP-Pe; SE-Pe; SE-RAr; RV-RP; SE-RV; VC-SE
PG (34:1)	9.13E-07	RP-Al; RV-Al; Pe-RAv; RP-RAv; RV-RAv; SE-RAv; VC-RAv; RF-Pe; RP-RF; RV-RF; SE-RF; VC-RF
DGTS (32:2)	1.43E-06	Pe-Al; Pe-RAv; RAr-RAv; RF-RAv; RV-Pe; SE-Pe; VC-Pe; SE-RAr; RV-RF; SE-RF; SE-RP
DGDG (36:5)	2.18E-06	RF-Al; RP-Al; VC-Al; Pe-RAv; RAr-RAv; RF-RAv; RP-RAv; VC-RAv; RV-RF; SE-RF; VC-RV; VC-SE
PI (34:3)	2.86E-06	Pe-Al; RAr-Al; VC-Al; RF-Pe; RP-Pe; SE-Pe; RF-RAr; RP-RAr; SE-RAr; VC-RF; VC-RP; VC-SE

SQDG (32:2)	3.67E-06	RV-Al; RF-RAv; RV-Pe; SE-Pe; RV-RAr; RP-RF; RV-RF; SE-RF; VC-RF
DGDG (34:8)	5.64E-06	RAv-Al; RV-Al; Pe-RAv; RF-RAv; RP-RAv; RV-Pe; RV-RF; RV-RP
SQDG (36:5)	5.79E-06	Pe-RAv; RF-RAv; RP-Pe; RV-Pe; SE-Pe; RP-RF; RV-RF; SE-RF
PC (32:3)	1.01E-05	Pe-Al; RAr-Al; RP-Al; VC-Al; RP-RAv; RP-RF; RV-RP; SE-RP
PC (34:5)	1.09E-05	RP-Al; RV-RAv; RV-Pe; RP-RAr; RP-RF; RV-RP; SE-RV
DGDG (34:2)	1.41E-05	RAv-Al; Pe-RAv; RAr-RAv; RF-RAv; RP-RAv; RV-RAv; VC-RAv
SQDG (32:1)	2.55E-05	RV-Al; SE-Al; RV-RAv; RF-Pe; RV-RAr; RP-RF; RV-RF; SE-RF; VC-RF
SQDG (36:2)	3.29E-05	RV-Al; SE-Al; RV-Pe; SE-Pe; SE-RAr; SE-RF; SE-RP; VC-SE
DGTS (36:2)	4.07E-05	Pe-Al; RAr-Al; RF-Pe; RV-Pe; RF-RAr; RV-RAr; SE-RAr; VC-RF
SQDG (34:2)	5.88E-05	RV-Pe; SE-Pe; RV-RAr; SE-RAr; RV-RP; SE-RP; VC-RV; VC-SE
MGMG (18:4)	5.99E-05	RAv-Al; Pe-Al; RF-Al; RV-Al; SE-Al; VC-Al; RV-RP
DGTS (34:1)	7.96E-05	Pe-RAv; RAr-RAv; RP-RAv; VC-RAv; RV-Pe; SE-Pe; RV-RAr; SE-RAr; SE-RF; SE-RP; VC-SE
PG (32:2)	8.87E-05	RP-Al; SE-Al; RP-RAv; SE-RAv; RP-RF; SE-RF
SQDG (34:4)	0.000105	RAr-Al; RV-Al; SE-Al; RV-RAv; SE-RAv; SE-Pe; RV-RF; SE-RF
LPC (16:0)	0.000112	RP-RAv; RP-RAr; RP-RF; SE-RP; SE-RV
DGDG (34:4)	0.000142	Pe-Al; Pe-RAv; RP-Pe; RV-Pe; SE-Pe
SQDG (33:1)	0.000265	RP-RAv; RV-RAv; SE-RAv; VC-RAv
PG (40:6)	0.000297	RV-Al; SE-Al; SE-RAv; SE-Pe; SE-RAr; SE-RF; SE-RP; VC-SE
SQDG (36:4)	0.000378	SE-Al; RF-RAv; SE-Pe; RV-RF; SE-RF; SE-RP
DGTS (32:3)	0.000409	Pe-RAv; RF-RAv; RP-RAv; VC-RAv; SE-Pe; SE-RP
SQDG (34:1)	0.0009	RV-Al; SE-Al; SE-RF
DGTS (30:1)	0.001223	RP-RAr; RP-RF; RV-RP; SE-RP
DGTS (32:1)	0.009099	RV-RF; VC-RF
SQDG (34:3)	0.009897	RP-RAv
DGDG (34:1)	0.016569	RF-RAv
MGDG (36:6)	0.017302	SE-Al; SE-Pe; SE-RF
PI (38:8)	0.021072	RP-RAv
PC (30:3)	0.022561	RAv-Al
PG (36:2)	0.033494	SE-RF
DGDG (34:3)	0.041392	RP-RAv

Table S5. Significant different lipid species of normalized areas of the top 25 lipid species. Normalized areas were obtained from extracted-ion chromatogram (XIC). Albufeira (Al), Ria de Aveiro (RAv), Peniche (Pe), Ria Arousa (RAr), Ria Formosa (RF), Ria de Pontevedra (RP), Ria de Vigo (RV), Sado Estuary (SE), and Viana do Castelo (VC). Significant difference ($p < 0.05$) among different locations are highlighted in grey shaded cells and black font numbers

Contrast		MGDG(32:8)	MGDG(32:3)	MGDG(32:0)	MGDG(34:7)	MGDG(36:9)	DGDG(36:3)	DGTS(28:0)	DGTS(40:8)	DGTS(40:7)	DGTS(40:6)	DGTS(40:5)	DGTS(42:11)	DGTS(42:9)	DGTS(44:10)	MGTS(18:0)	MGTS(22:0)	PC(30:0)	PC(38:8)	PC(40:10)	PC(40:7)	LPE(22:5)	PE(40:9)	SQDG(28:0)	SQDG(30:1)	LPG(16:1)
Al	RAv	<0.0001	<0.0001	<0.0001	<0.0001	<0.0001	<0.0001	<0.0001	<0.0001	<0.0001	<0.0001	<0.0001	<0.0001	<0.0001	<0.0001	0.0321	<0.0001	<0.0001	<0.0001	<0.0001	<0.0001	<0.0001	<0.0001	<0.0001	<0.0001	<0.0001
	Pe	<0.0001	<0.0001	<0.0001	<0.0001	0.4242	<0.0001	<0.0001	0.0844	<0.0001	<0.0001	<0.0001	<0.0001	<0.0001	0.9973	<0.0001	<0.0001	1.0000	<0.0001	<0.0001	1.0000	<0.0001	0.9779	<0.0001	<0.0001	<0.0001
	RAr	1.0000	0.1341	<0.0001	<0.0001	<0.0001	0.0001	0.9605	0.1393	0.2255	0.9758	<0.0001	<0.0001	<0.0001	<0.0001	<0.0001	<0.0001	<0.0001	<0.0001	<0.0001	<0.0001	<0.0001	0.7009	<0.0001	<0.0001	<0.0001
	RF	<0.0001	<0.0001	0.0396	0.9998	<0.0001	<0.0001	<0.0001	<0.0001	<0.0001	<0.0001	<0.0001	<0.0001	0.0900	<0.0001	<0.0001	<0.0001	<0.0001	<0.0001	<0.0001	0.7687	<0.0001	0.0093	<0.0001	<0.0001	<0.0001
	RP	0.0129	<0.0001	<0.0001	<0.0001	<0.0001	<0.0001	<0.0001	<0.0001	<0.0001	<0.0001	<0.0001	<0.0001	<0.0001	0.2638	<0.0001	<0.0001	<0.0001	<0.0001	<0.0001	<0.0001	<0.0001	<0.0001	<0.0001	<0.0001	<0.0001
	RV	<0.0001	<0.0001	<0.0001	0.1469	<0.0001	<0.0001	<0.0001	<0.0001	<0.0001	<0.0001	1.0000	0.0006	<0.0001	0.0001	<0.0001	<0.0001	0.7353	<0.0001	<0.0001	0.5332	<0.0001	<0.0001	<0.0001	<0.0001	<0.0001
	SE	<0.0001	<0.0001	<0.0001	<0.0001	<0.0001	<0.0001	<0.0001	<0.0001	<0.0001	0.0001	<0.0001	<0.0001	<0.0001	<0.0001	<0.0001	0.6051	<0.0001	<0.0001	<0.0001	<0.0001	<0.0001	<0.0001	<0.0001	0.0009	<0.0001
	VC	<0.0001	<0.0001	<0.0001	<0.0001	<0.0001	<0.0001	<0.0001	1.0000	0.0004	0.9980	<0.0001	<0.0001	<0.0001	<0.0001	<0.0001	<0.0001	<0.0001	<0.0001	<0.0001	<0.0001	<0.0001	0.0328	0.0057	<0.0001	<0.0001
RAv	Pe	<0.0001	<0.0001	<0.0001	<0.0001	<0.0001	<0.0001	<0.0001	<0.0001	<0.0001	<0.0001	<0.0001	<0.0001	<0.0001	<0.0001	<0.0001	0.0002	<0.0001	<0.0001	<0.0001	<0.0001	<0.0001	<0.0001	<0.0001	<0.0001	<0.0001
	RAr	0.0001	<0.0001	0.0008	<0.0001	<0.0001	<0.0001	<0.0001	<0.0001	<0.0001	<0.0001	<0.0001	<0.0001	<0.0001	<0.0001	<0.0001	<0.0001	<0.0001	<0.0001	<0.0001	0.0004	<0.0001	<0.0001	<0.0001	1.0000	<0.0001
	RF	0.0001	<0.0001	<0.0001	<0.0001	<0.0001	0.0001	0.1681	<0.0001	<0.0001	<0.0001	<0.0001	<0.0001	<0.0001	<0.0001	<0.0001	<0.0001	1.0000	<0.0001	<0.0001	<0.0001	<0.0001	<0.0001	0.0122	<0.0001	<0.0001
	RP	0.0001	<0.0001	<0.0001	<0.0001	<0.0001	<0.0001	<0.0001	<0.0001	<0.0001	<0.0001	<0.0001	<0.0001	<0.0001	<0.0001	0.6543	<0.0001	<0.0001	<0.0001	1.0000	<0.0001	<0.0001	1.0000	<0.0001	<0.0001	<0.0001
	RV	0.0001	<0.0001	<0.0001	<0.0001	<0.0001	<0.0001	<0.0001	<0.0001	<0.0001	<0.0001	<0.0001	<0.0001	<0.0001	<0.0001	<0.0001	<0.0001	<0.0001	<0.0001	0.0001	<0.0001	<0.0001	<0.0001	<0.0001	<0.0001	<0.0001
	SE	0.0001	<0.0001	<0.0001	<0.0001	<0.0001	<0.0001	1.0000	1.0000	0.0001	0.9997	<0.0001	<0.0001	0.1076	<0.0001	<0.0001	<0.0001	0.0338	<0.0001	0.0001	0.5616	1.0000	1.0000	<0.0001	<0.0001	1.0000
	VC	<0.0001	<0.0001	<0.0001	<0.0001	<0.0001	<0.0001	<0.0001	<0.0001	<0.0001	<0.0001	<0.0001	<0.0001	<0.0001	<0.0001	<0.0001	<0.0001	<0.0001	<0.0001	<0.0001	0.7933	<0.0001	<0.0001	0.2740	0.9183	<0.0001
Pe	RAr	<0.0001	<0.0001	<0.0001	<0.0001	<0.0001	0.0084	<0.0001	<0.0001	<0.0001	<0.0001	0.9926	<0.0001	<0.0001	<0.0001	<0.0001	<0.0001	<0.0001	<0.0001	0.9912	<0.0001	0.9980	0.9986	<0.0001	<0.0001	0.9980
	RF	0.0289	<0.0001	<0.0001	<0.0001	<0.0001	<0.0001	<0.0001	<0.0001	<0.0001	<0.0001	<0.0001	<0.0001	<0.0001	<0.0001	<0.0001	0.1156	<0.0001	0.2872	<0.0001	0.7244	<0.0001	0.0003	<0.0001	<0.0001	0.0001
	RP	<0.0001	<0.0001	1.0000	<0.0001	<0.0001	<0.0001	1.0000	0.2305	<0.0001	0.2547	<0.0001	0.1035	0.0001	0.7539	<0.0001	<0.0001	<0.0001	<0.0001	<0.0001	<0.0001	0.4651	<0.0001	1.0000	<0.0001	0.4651
	RV	<0.0001	<0.0001	<0.0001	<0.0001	<0.0001	<0.0001	<0.0001	<0.0001	<0.0001	<0.0001	<0.0001	<0.0001	<0.0001	<0.0001	<0.0001	0.9949	0.7045	0.1672	0.7702	0.4839	<0.0001	<0.0001	<0.0001	<0.0001	<0.0001
	SE	<0.0001	<0.0001	0.4334	<0.0001	<0.0001	<0.0001	<0.0001	<0.0001	<0.0001	<0.0001	0.0001	<0.0001	<0.0001	<0.0001	<0.0001	<0.0001	<0.0001	<0.0001	<0.0001	<0.0001	<0.0001	<0.0001	1.0000	0.8420	<0.0001
	VC	<0.0001	<0.0001	<0.0001	<0.0001	<0.0001	0.0003	<0.0001	0.2249	0.0992	<0.0001	0.4954	<0.0001	0.0005	<0.0001	<0.0001	<0.0001	<0.0001	<0.0001	<0.0001	<0.0001	<0.0001	0.0013	<0.0001	<0.0001	<0.0001
RAr	RF	<0.0001	<0.0001	<0.0001	<0.0001	<0.0001	<0.0001	<0.0001	<0.0001	<0.0001	<0.0001	<0.0001	<0.0001	<0.0001	<0.0001	<0.0001	<0.0001	<0.0001	<0.0001	<0.0001	<0.0001	<0.0001	<0.0001	<0.0001	<0.0001	<0.0001
	RP	0.0140	<0.0001	<0.0001	<0.0001	<0.0001	<0.0001	<0.0001	<0.0001	<0.0001	0.0479	<0.0001	<0.0001	0.6444	<0.0001	<0.0001	<0.0001	<0.0001	<0.0001	<0.0001	<0.0001	0.1041	<0.0001	<0.0001	<0.0001	0.1041
	RV	<0.0001	<0.0001	<0.0001	<0.0001	<0.0001	<0.0001	0.0002	<0.0001	<0.0001	<0.0001	<0.0001	<0.0001	<0.0001	0.6012	0.9990	<0.0001	<0.0001	<0.0001	0.9983	<0.0001	<0.0001	<0.0001	<0.0001	<0.0001	<0.0001
	SE	<0.0001	<0.0001	<0.0001	<0.0001	<0.0001	<0.0001	<0.0001	<0.0001	<0.0001	<0.0001	<0.0001	<0.0001	<0.0001	<0.0001	<0.0001	<0.0001	<0.0001	<0.0001	<0.0001	<0.0001	<0.0001	<0.0001	<0.0001	<0.0001	<0.0001
	VC	<0.0001	<0.0001	<0.0001	<0.0001	1.0000	0.9922	<0.0001	0.0467	0.4016	1.0000	0.9611	0.0030	0.3328	0.9278	0.7807	<0.0001	<0.0001	<0.0001	<0.0001	<0.0001	<0.0001	<0.0001	<0.0001	0.8623	<0.0001
RF	RP	<0.0001	<0.0001	<0.0001	<0.0001	<0.0001	<0.0001	<0.0001	<0.0001	<0.0001	<0.0001	<0.0001	<0.0001	<0.0001	<0.0001	<0.0001	0.0126	<0.0001	<0.0001	<0.0001	<0.0001	<0.0001	<0.0001	<0.0001	<0.0001	<0.0001
	RV	<0.0001	<0.0001	<0.0001	0.0341	<0.0001	<0.0001	0.0426	<0.0001	0.0001	0.0338	<0.0001	<0.0001	0.0007	0.0016	<0.0001	0.5370	<0.0001	1.0000	<0.0001	1.0000	<0.0001	<0.0001	<0.0001	<0.0001	<0.0001
	SE	<0.0001	1.0000	<0.0001	<0.0001	<0.0001	0.9399	0.2787	<0.0001	<0.0001	<0.0001	<0.0001	<0.0001	<0.0001	<0.0001	<0.0001	<0.0001	0.0119	<0.0001	<0.0001	<0.0001	<0.0001	<0.0001	<0.0001	<0.0001	<0.0001
	VC	<0.0001	<0.0001	<0.0001	<0.0001	<0.0001	<0.0001	<0.0001	0.0001	<0.0001	<0.0001	<0.0001	<0.0001	<0.0001	<0.0001	<0.0001	<0.0001	<0.0001	<0.0001	<0.0001	<0.0001	<0.0001	1.0000	<0.0001	<0.0001	<0.0001
RP	RV	<0.0001	<0.0001	<0.0001	<0.0001	<0.0001	<0.0001	<0.0001	<0.0001	<0.0001	<0.0001	<0.0001	<0.0001	<0.0001	<0.0001	<0.0001	<0.0001	<0.0001	<0.0001	<0.0001	<0.0001	<0.0001	<0.0001	<0.0001	<0.0001	<0.0001
	SE	<0.0001	<0.0001	0.3125	<0.0001	<0.0001	<0.0001	<0.0001	<0.0001	<0.0001	<0.0001	<0.0001	<0.0001	<0.0001	<0.0001	<0.0001	<0.0001	<0.0001	<0.0001	<0.0001	<0.0001	<0.0001	1.0000	1.0000	<0.0001	<0.0001
	VC	<0.0001	<0.0001	<0.0001	0.0009	<0.0001	<0.0001	<0.0001	<0.0001	<0.0001	0.0187	<0.0001	0.0015	0.9999	<0.0001	<0.0001	<0.0001	1.0000	<0.0001	<0.0001	<0.0001	<0.0001	<0.0001	<0.0001	<0.0001	<0.0001
RV	SE	<0.0001	<0.0001	<0.0001	0.0044	0.7784	<0.0001	<0.0001	<0.0001	<0.0001	<0.0001	<0.0001	<0.0001	<0.0001	<0.0001	<0.0001	<0.0001	<0.0001	<0.0001	<0.0001	<0.0001	<0.0001	<0.0001	<0.0001	<0.0001	<0.0001
	VC	1.0000	<0.0001	0.8045	<0.0001	<0.0001	<0.0001	<0.0001	<0.0001	<0.0001	<0.0001	<0.0001	<0.0001	<0.0001	0.0439	0.9887	<0.0001	<0.0001	<0.0001	<0.0001	<0.0001	<0.0001	<0.0001	<0.0001	<0.0001	<0.0001
SE	VC	<0.0001	<0.0001	<0.0001	<0.0001	<0.0001	<0.0001	<0.0001	<0.0001	<0.0001	<0.0001	<0.0001	<0.0001	<0.0001	<0.0001	<0.0001	<0.0001	<0.0001	<0.0001	<0.0001	1.0000	<0.0001	<0.0001	<0.0001	<0.0001	<0.0001
